# Phase Stability and Electronic Properties of Hybrid Organic–Inorganic Perovskite Solid Solution (CH(NH_2_)_2_)_*x*_(CH_3_NH_3_)_1–*x*_Pb(Br_*y*_I_1–*y*_)_3_ as a Function of Composition

**DOI:** 10.1021/acs.jpcc.2c03555

**Published:** 2022-08-04

**Authors:** T. H. Chan, N. T. Taylor, S. Sundaram, S. P. Hepplestone

**Affiliations:** † Department of Physics and Astronomy, College of Engineering, Mathematics and Physical Sciences, Streatham Campus, University of Exeter, Exeter EX4 4QL, U.K.; ‡ The School of Engineering and the Built Environment, Merchiston Campus, Edinburgh Napier University, Edinburgh EH10 5DT, U.K.

## Abstract

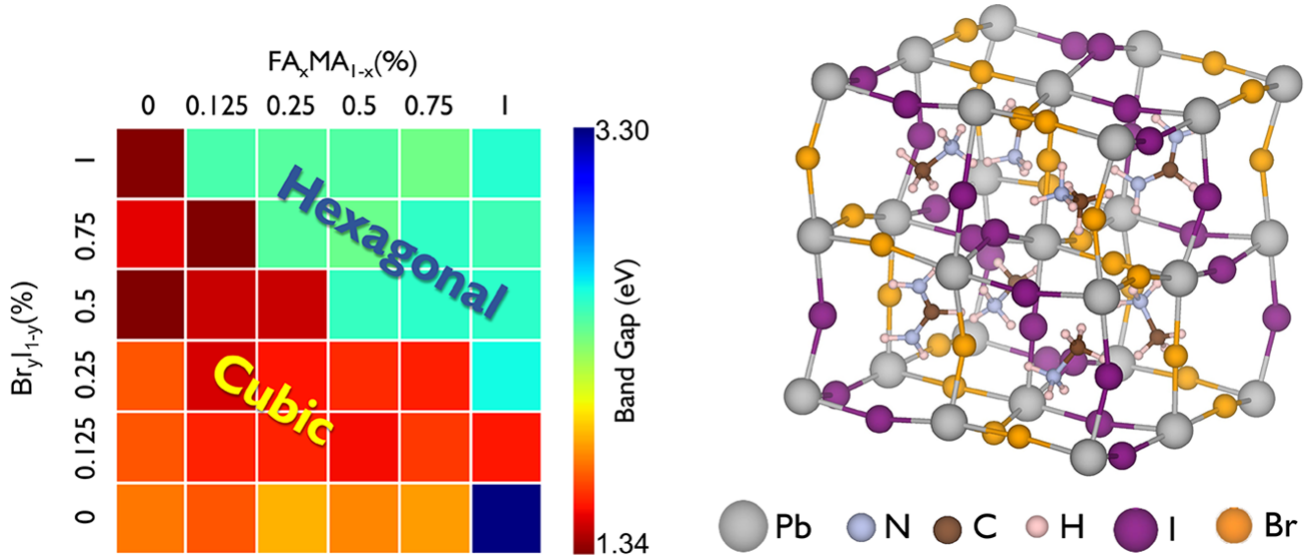

Compositional mixing provides the means to maintain the structural stability of a hybrid organic–inorganic perovskite for efficient and robust photovoltaic applications. Here we present a theoretical, first-principles study of the electronic and energetic properties of the solid solution (CH(NH_2_)_2_)_*x*_(CH_3_NH_3_)_1–*x*_PbBr_*y*_I_1–*y*_, the mixing of two organic molecules with various orientations, formamidinium and methylammonium, and two halides, bromide and iodide. Our results show the variation in the band gap as a function of composition (*x* and *y*) provides several candidates that exceed the 27.5% Schockley–Queisser efficiency. The variation in the composition of hybrid perovskite shows specific regions where either the hexagonal or cubic phase dominates. We discuss the balance between the band gap and phase stability and indicate regions where the phase transition temperature between cubic and hexagonal phases is far from room temperature, indicating that these compositions are more robust at room temperature against phase transitions.

## Introduction

Hybrid organic–inorganic perovskites have potential for various optoelectronic applications,^1,2^ especially as solar PV materials, as they have demonstrated a higher electrical power conversion efficiency (PCE) than the commercial polycrystalline silicon solar module (22%).^3^ Since 2009, the performance of these hybrid perovskites has increased from 3.8%^4^ to 25.2%.^5^ According to the Shockley–Queisser limit, materials with band gaps of 0.9–1.75 eV can potentially possess PCEs of >27.5%, which is ideal for a solar cell.^6^ Alternatively, wider band gap materials can be considered for use in tandem solar cells. Hybrid perovskites exhibit a range of values for their band gaps, depending on composition and temperature. Therefore, they have more significant potential to be more suitable than the well-developed, conventional crystalline silicon solar cell (1.1 eV band gap^7^) practically and theoretically. In particular, recent advances have focused on mixed hybrid perovskites due to their better stability and performance.^8,9^ These hybrid perovskites have a general formula of ABX_3_, where A, B, and X are specifically an organic molecule, a post-transition metal, and a halide, respectively. The most notable of these is methylammonium lead iodide (MAPI), with a band gap of 1.55 eV^10,11^ and a reported efficiency of 20.4%.^12^

Initial investigations on inorganic tin perovskite and hybrid organic–inorganic tin perovskites by Borriello et al.^13^ reported that the geometrical structure and crystal phase were influenced by the size and orientation of the embedded organic molecule, that is, methylammonium (MA). This, in turn, impacts the resultant electronic properties. In particular, the band gap of these compounds is heavily affected by the deformation and breaking of the inorganic cage due to the organic molecule.^14,15^ Experimental studies showed MAPI generally has a band gap of 1.5–1.6 eV, low charge carrier recombination rates, low effective masses,^16^ high charge carrier mobilities (35 cm^2^ V^–1^ s^–1^),^17^ and high optical absorption.^18^ These initial properties lead to a PCE that is comparable to polycrystalline silicon.^19^ However, the measured efficiencies of these devices varied considerably depending on fabrication,^12,20,21^ indicating instability of the crystal. Yang et al.^22^ showed that MAPI degraded in water, and the process was more rapid under light conditions. They identified reaction intermediates, such as isolated PbI_6_^4–^ and hydrate [CH_3_NH_3_]_4_PbI_6_·H_2_O which occurred during the degradation. The degradation results come from photogenerated holes promoting iodine vacancies,^23,24^ and these vacancies lower the energy barrier for the water to dissociate. H^+^ and OH^–^ ions then react with iodine on the surface and the organic molecules with the aid of a photogenerated electron, forming a vicious cycle leading to degradation of MAPI. This instability was resolved by including small amounts of Cs into the hybrid organic.^25^ However, the effect of including Cs is to generally increase the band gap (as CsPbI_3_ has a band gap of 1.73 eV^26−30^).

To improve the properties of the hybrid perovskite, by both reducing the optical gap and increasing the water stability, mixed systems are now the subject of intense investigation.^31−33^ However, these mixed systems then have further issues due to phase stability. For band gap manipulation, mixing formamidinium^34^ with methylammonium for the A site is a strong candidate as FAPI has a lower band gap of 1.43 eV.^35^ However, FAPI at room temperature tends to form the hexagonal δ phase.^36^ This is further complicated by MAPI showing changes from tetragonal β phase to cubic α phase at 327 K, slightly higher than room temperature.^37−39^ The concern about the temperature means that during use heating effects (from solar absorption) cause the film to undergo a phase transition which reduces the effectiveness of the cell. For the X site, Mosconi et al.^32^ demonstrated that changing the compositions of the halide provides better structural stability during the formation due to less variation in size of I or Br compared to the MA molecule. However, a greater degree of distortion was observed when Cl was introduced rather than I or Br. Further studies^40^ showed that the ordered distribution of Br and I was generally more stable than the disordered. However, an optimum temperature for fabrication of 343 K was required for uniform distribution of the halides. This mixing was shown^41^ to help to maintain the cubic phase at room temperatures. However, the formation of MAPB results in a band gap of 2.2 eV.^42^

In this study, we focus on investigating the interplay between mixing the A and X sites, considering formamidinium (FA) and methylammonium (MA) as well as Br and I to explore what combinations promote cubic stability compared to the hexagonal δ phase and how this affects the subsequent electronic properties. We adopt a computational approach to investigate the microscopic properties of the mixing between the A and X sites in hybrid perovskites. The resulting chemical composition is FA_*x*_MA_1–*x*_Pb[Br_*y*_I_1–*y*_]_3_ where *x* and *y* are between 0 and 1 and MA and FA have their usual chemical formulas, CH_3_NH_3_^+^ and CH(NH_2_)_2_^+^, respectively. We also consider the effects of organic molecular alignment and how this can influence the phase of the system. From this, we explore how the optical gap and the relative stability of the two phases change for various compositions at different temperatures. This helps establish the role of individual chemical components and their combining effect to construct an efficient thin-film solar PV material.

## Computational Method

The study of the electronic and energetic properties was performed by using the Vienna Ab Initio Simulation Package (VASP).^43,44^ VASP utilizes a plane-wave basis set by using the projector-augmented wave (PAW) pseudopotentials. For structural optimization, the Generalized Gradient Approximation (GGA) is used for the exchange-correlation potential with a PBE framework.^45^ We do not include spin–orbit coupling, as this results in vastly underestimated band gaps (see Table S8), which requires an additional hybrid correction to counter. Instead, we take full advantage of this fortuitous cancellation of errors so that our calculations can consider large supercells beyond the typical range of hybrid calculations.^46^ Because organic molecules are present, D3 correction was also applied for the van der Waals interaction.^47,48^ The results from PBE functional and van der Waals corrections showed accurate band gaps in comparison to experimental data. The details are discussed in Table S8. We use a plane-wave energy cutoff of 550 eV and a 3 × 3 × 3 *k*-point grid^49^ for both the cubic and hexagonal structures. The elements in the system include C, H, I, Br, N, and Pb. C and Pb each have a valency of 2s^2^2p^2^ and 6s^2^6p^2^, respectively. The halides have a valency of 4s^2^4p^5^ for Br and 5s^2^5p^5^ for I, respectively. H has 1s^1^, and N has a valency of 2s^2^2p^3^. All structures are relaxed by using the conjugate gradient algorithm. The structures are considered relaxed during the relaxation calculations when the maximum force acting on any atom is <5 meV/Å.

**Figure 1:**
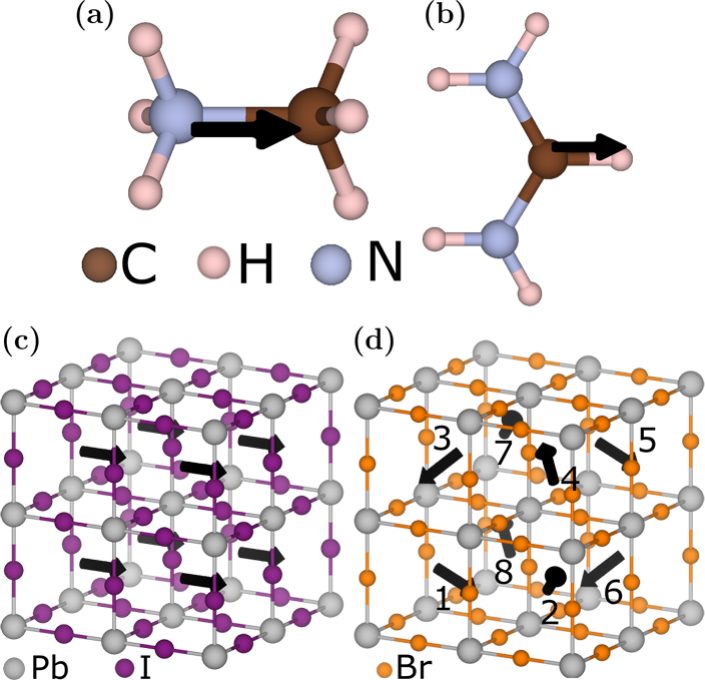
Schematic structure of the (a) MA and (b) FA molecule. (c) A 2 × 2 × 2 supercell with aligned molecules with respect to each other whereas (d) shows another supercell with symmetrically rotated molecules according to Table S1. The arrows in both figures indicate the orientation of the molecules. Gray, purple, and yellow atoms are Pb, I, and Br, respectively.

To model the properties of FA_*x*_MA_1–*x*_Pb[Br_*y*_I_1–*y*_]_3_, we considered a 2 × 2 × 2 supercell. Each supercell can be considered to have subunits of methylammonium lead iodide (MAPI), methylammonium lead bromide (MAPB), formamidinium lead iodide (FAPI), or formamidinium lead bromide (FAPB) depending on its stoichiometry. Details of lattice parameters and atomic positions are shown in Tables S3–S6. Thus, by varying the 8 A and 24 X sites in each supercell, we can examine the properties of the various chemical compositions. Some compositions can have multiple permutations, In the 2 × 2 × 2 supercell, we considered all possible permutations by swapping unit cells of the constituents and switching between tribromide and triiodide and between MA and FA molecules within the unit cell. The organic molecule structures are shown in Figure 1a (MA) and Figure 1b (FA). These systems are known to form multiple phases, with hexagonal and cubic being the most electronically distinct. In theory, the hexagonal 2H phase is the preferable phase for 3D hybrid perovskites at zero temperature condition.^50,51^ However, this has not been proven in experimental studies. Instead, there is a transition of MAPI from the tetragonal to cubic phase,^52^ whereas the transition of FAPI is from cubic to 2H hexagonal phase^36^ around room temperature. To understand the potential phase transition, that is, the stability, both 2H hexagonal and cubic variations are considered in all hybrid perovskites.

**Figure 2:**
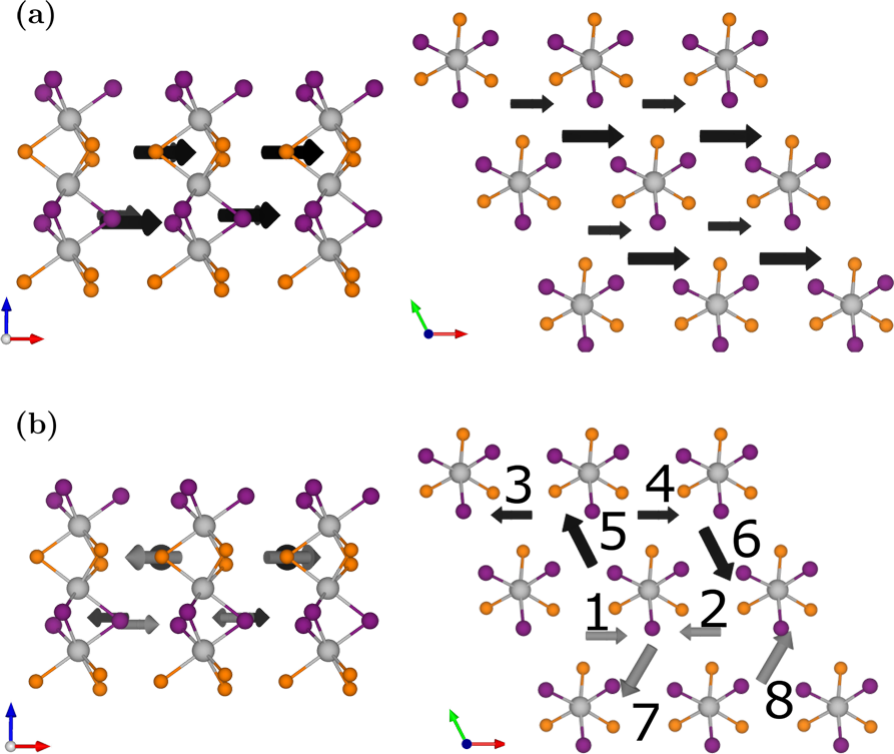
(a) A 2 × 2 × 2 hexagonal supercell with aligned molecules with respect to each other whereas (b) shows the cubic perovskite with symmetrically rotated molecules according to Table S2. The arrows in both figures indicate the orientation of the molecules. Gray, purple, and yellow atoms are Pb, I, and Br, respectively.

In the context of the cubic supercell, the constituents’ in-plane lattice parameters and angles are within a tolerance of 1% difference and 5°, respectively. Thus, the lattice-matched supercells are technically orthorhombic or tetragonal but here are referred to as “cubic-like” due to the constituents from which they are made. Details of the differences between the cubic-like phases are discussed in the Supporting Information. Examples of cubic-like supercells are shown in Figures 1c and 1d. Each cubic-like supercell consists of eight diamond-shaped PbX_6_ polyhedra where Pb is at the center, bonded with six halide atoms (Br or I). Arrows situated between polyhedra represent the aforementioned organic molecules with the corresponding orientation. Similarly, an example of the hexagonal supercell is FA_0.5_MA_0.5_Pb[Br_0.5_I_0.5_]_3_, shown in Figures 2a and 2b. The supercell contains sliding chains of hourglass-shaped PbX_6_, where Pb is situated at the center, connecting two trihalide planes.

The orientation of the molecules has a direct influence on the properties of the hybrid perovskites, so we consider multiple configurations of the organic molecule. Because of the composition, we define the molecular orientation based on its polarization (δ+ and δ−). For the bulk FAPI and MAPI structures, Sukmas et al.^53^ reported 343 orientations varying the three Euler angles and found the highest energy barrier to FA molecules to be 25 meV per chemical unit, meaning that they are freely rotating at room temperature. Furthermore, Quarti et al.^54^ showed the molecular orientation configuration in the experimental sample could be modeled as the thermal average of different simulated configurations. Following their works, discussed in the Supporting Information, we examined the various orientations of MA and FA molecules in MAPI, MAPB, FAPI, and FAPB each in the cubic-like phase. They showed that most permutations of the FA molecule are not favorable by up to 0.3 eV per chemical unit, given that the thermal excitation at room temperature is only 26 meV per chemical unit. On the basis of these results, for examining the mixed composition space, we have reduced the number of potential configurations for the organic alignment to two, which we refer to as aligned and unaligned. This study focused on the phase stability instead of the variation of the molecular orientations. We note that our unaligned cell contains deliberately four distinct orientations, capturing several extrema positions in the spirit of the approach by Quarti et al.^54^ Moreover, the nature of uneven interaxial angles of the hexagonal phase restricts molecular rotations. The permutations are shown in Figures 1c and 1d for the cubic-like phase and Figures 2a and 2b for the hexagonal phase. Considering further orientations in combination with a large number of permutations of the A and X sites was beyond the feasibility of this study due to the large size of the supercells used. The combination of orientations and permutations of the mixture provides an effective sample range for each stoichiometry, allowing us to assess the variance in the properties as discussed below.

Phase stability is determined via evaluation of the Gibbs free energy. Evaluations of this requires calculations of the phonon modes of the system. For most systems, the contribution from the vibrational modes is small, but for hybrid perovskites, the relative difference in the phase stability is comparable with the vibrational term. Thus, the phonon modes of the system need to be evaluated. Our bulk constituents are relaxed to reach threshold of 1 meV/Å, and the phonon frequencies are obtained by using the The PHONOPY approach.^55^ Following this procedure, a comparison of the enthalpy of two different phases (cubic-like and hexagonal) at temperature, *T*, can be made, taking into account the vibrational entropy. The Gibbs free energy is evaluated following the approach set out by Butler et al.^38^ and Wei et al.^56^ The difference in the Gibbs free energy, Δ*G*, between the two phases is expressed as
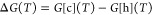
1where *G*[c/h](*T*) indicates the Gibbs free energy of the cubic-like or hexagonal phase, respectively. This Gibbs free energy is given as
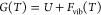
2where *U* is the internal energy and *F*_vib_(*T*) is the vibrational free energy. The vibrational energy is further expanded as
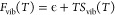
3where ϵ is zero point vibrational energy, given as ϵ = ℏω. The vibrational entropy, *S*_vib_(*T*) (in eV/K), is
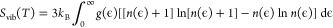
4where *k*_B_ is the Boltzmann constant, *g*(ϵ) is the normalized phonon density of states with energy ϵ, *n*(ϵ) is the Bose–Einstein population of a state of energy ϵ at temperature *T*, and ϵ = ℏω, where ω is the phonon mode frequency. Calculating all the vibrational modes of our mixed system supercells is beyond current computational limits. To evaluate the stability of the supercells, it is assumed that the entropy contributions are proportional to the bulk constituents. While the changes to the entropy due to the presence of an interface between constituents are likely, these contributions will be small (<0.01 meV). Such an approximation is well-justified^57−60^ due to most vibrational modes being dominated by nearest-neighbor interactions.

Besides evaluating the preferable phase of the mixed hybrid perovskites, one has to assess its relative stability compared to its constituents in the same phase. Such energy provides insight into the likelihood of the mixed system being uniformly mixed instead of having separate segregated regions of differing compositions. We calculate this by using the following formation energy
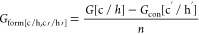
5where *G*_form[c,c′]_ is the formation energy of the cubic-like structure with respect to cubic-like constituents in eV (per chemical unit), *G*_form[h,h′]_ is the formation energy of the hexagonal structure with respect to hexagonal constituents in eV (per chemical unit), and *n* is the total number of chemical units. *G*_con_ of FA_*x*_MA_1–*x*_Pb[Br_*y*_I_1–*y*_]_3_ is given as
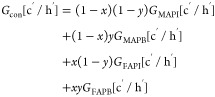
6where *G*_MAPI_, *G*_MAPB_, *G*_FAPI_, and *G*_FAPB_ are the Gibbs free energies of the geometrically optimized MAPI, MAPB, FAPI, and FAPB, respectively, including the entropy term. At 0 K, this approach shows the hexagonal phase of MAPI and FAPI is the most energetically preferable, in agreement with Thind et al.^50^ and Chen et al.,^51^ respectively. Also, our calculations show that, at 0 K, FAPB adopts the hexagonal phase and MAPB adopts the cubic-like phase.

## Results

**Figure 3:**
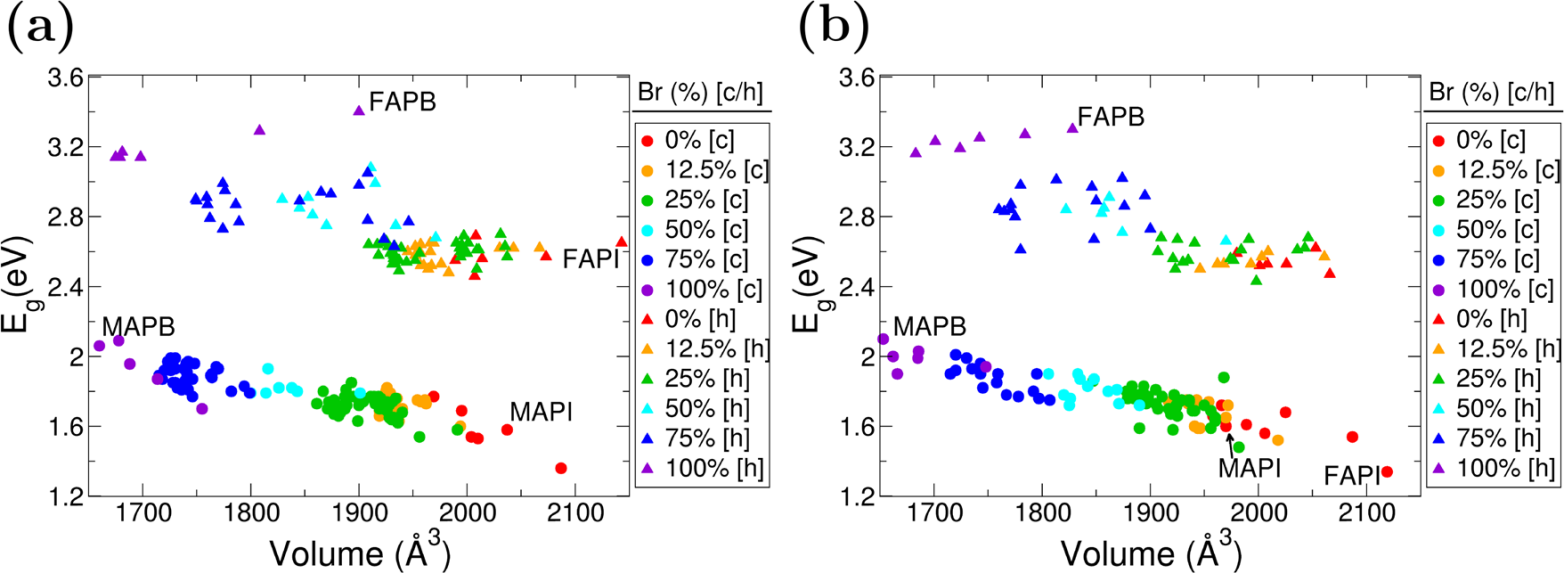
Correlation between volume and band gap of the hexagonal or cubic-like FA_*x*_MA_1–*x*_Pb[Br_*y*_I_1–*y*_]_3_ hybrid perovskite supercell with the arrangement of (a) aligned and (b) rotated organic molecules. The colors differentiate the proportion of Br to I. The MA–FA mixture is not specified as it shows no clear trend.

The electronic band gap is generally determined by the phase and composition of the material (though other factors can have an influence^39,61^). The results presented in Figure 3 show distinctive segregation of the band gap of perovskites between hexagonal and cubic-like phases as expected, with the hexagonal phase exhibiting a higher band gap than the cubic-like phase. The hexagonal phase band gaps range from 2.4 to 3.3 eV near the UV spectrum, whereas the band gaps exhibit the lower band gap ranging from 1.4 to 2.0 eV. The electronic characteristics in the region of the band gap are dominated by the Pb and halides. In both phases, the Pb is the dominant contributor to the CBM states, whereas the VBM is a mixture of Pb and the halide.

All-perovskite tandem cells have been previously proposed,^62^ and here we indicate how manipulating the two phases could lead to suitable candidates. Candidates with high amounts of FA (>75%) and <25% of Br would be ideal for absorbing high-frequency photons in a tandem solar cell. Our remaining discussion will focus on the more challenging low band gap component. Our results in Figure 3 suggest that for the cubic-like phase with 50% iodine composition or higher all mixes will result in a Shockley–Quëisser limit of 27.5% or higher, comparable to the efficiency of silicon.

The band gap difference between these two phases can be explained as being a result of the difference in the polarization of the two structures. We analyze this using Bader charge analysis.^63^ The details are discussed in Table S9.

For MAPI, the cubic-like phase can be described as a (CH_3_NH_3_)I layer and a PbI_2_ layer, each with a total charge of ±0.1 |*e*|. In comparison, the hexagonal phase can be described in two different ways, depending on orientation. It can be thought of as either PbI_3_ and (CH_3_NH_3_) layers or Pb and (CH_3_NH_3_)I_3_ layers. Both result in much more significant polarization differences between the layers, which are greater than 0.5 |*e*| in magnitude (in either direction). Thus, while the difference in Bader charges of individual atoms between phases is negligible (changes of <0.03 |*e*|), the strongly polarized layers of hexagonal perovskite result in a high band gap. This conclusion is further supported by considering that the volumetric difference remains insignificant (up to 4%) in hybrid perovskites between two phases. However, as expected, the unit cell of the hexagonal phase has a significant asymmetry. This asymmetry means the hexagonal unit cell has a larger cross-sectional area by +60% and a shorter perpendicular axis of −37%, whereas the three lattice parameters of cubic-like unit cell are approximately the same. This polarization difference is observed in general across the data set.

In the solid solution of FA_*x*_MA_1–*x*_Pb[Br_*y*_I_1–*y*_]_3_, the variation of the band gap with its constituents could be expected to follow Vegard’s law.^64^ As shown in Figure 3, for the I and Br, this law is relatively well obeyed, but the combination of rotational effects and various permutations leads to a spread in the energy of ≈0.3 eV. Figure 3 shows a decreasing linear trend between band gap and volume where the higher volume perovskites exhibit lower band gaps. This volume is determined by the ratio of Br–I, and hence so is the overall band gap of the structures, in both cubic-like and hexagonal phases. The increased volume of the structure results in a weaker Coulomb interaction between Pb and halides. As expected, the lowest band gap, 1.34 eV, and largest unit cell volume are found for FAPI in cubic-like phase. There is no correlation between the A site’s choice of organic (MA and FA), its band gap, and volume (see Figures S10a and S10b) because the volume is predominantly determined by the halide–lead distance. This is in agreement with Meloni et al.,^65^ who noted a similar behavior for mixed systems where the A site consisted of Li, Na, Cs, and other rotationally invariant cations. In our study, the effect of molecular orientation is found to be negligible as the small changes in volume due to changes in the organic molecule are generally compensated for by the rotation of the molecule in adjacent unit cells. As a result, it minimizes the resulting strain effects of changing the organic. For a fixed stoichiometry, the band gap variation is typically of the order of 0.3 eV for both the cubic-like phase and the hexagonal phase. Two factors drive this spread, including molecular orientations and the various permutations within the same stoichiometry.

Theoretical considerations of hybrid perovskites must consider the role of molecular orientation effects of the organic molecule. In alloys, this effect is further complicated by the relative distribution of the organics for any single solid solution composition. For fixed chemical composition, and comparing our two molecular orientation configurations for the perovskites (see Figure 3a,b), we show that for the general case halide composition is the more critical factor for determining the band gap than the choice of organic. By comparison of the perovskites of aligned and rotated molecules in Figure 3a,b, the volumetric changes are up to 4.2% in the hexagonal phase, whereas the change in cubic-like phase is insignificant (<1%). With this consideration, we have examined how much our two configurations for the organic molecules change the band gap. For the hexagonal phase, the result is a spread in the electronic gaps of 0.3 eV, particularly compositions with >50% Br. For cubic-like phases, the spread is slightly smaller, up to 0.2 eV. For bulk MAPI, our results show that the unit cell volume decreases when the molecules are given differing orientations (compared to the align system), but the band gap increases. This behavior differs from the effect of strain, which shows that (for a fixed configuration of organics) the band gap increases with increasing unit cell volume. Thus, for the mixed system, the change in the electronic gap is mainly driven by the I:Br ratio. Quarti et al.^54^ showed for cubic MAPI that the effect of rotating various methylammonium molecules was to create a spread in the band gaps of 0.2 eV but fix the unit cell volume. In our case, the unit cell total volume was allowed to fluctuate with a change in molecular orientation but achieved a similar spread in band gaps. These results agree that the chief contributor to this spread is the orientation, not local volume changes. This is further supported by examining the tilt angle of the polyhedra (see Figure S14b) which shows that the tilt of the polyhedra is increased for increasing amounts of methylammonium (and decreasing amounts of formamidinium). Hence, one can state that the band gap is determined by the I:Br ratio, but the spread is determined by the rotation/orientation of the organics.

**Figure 4:**
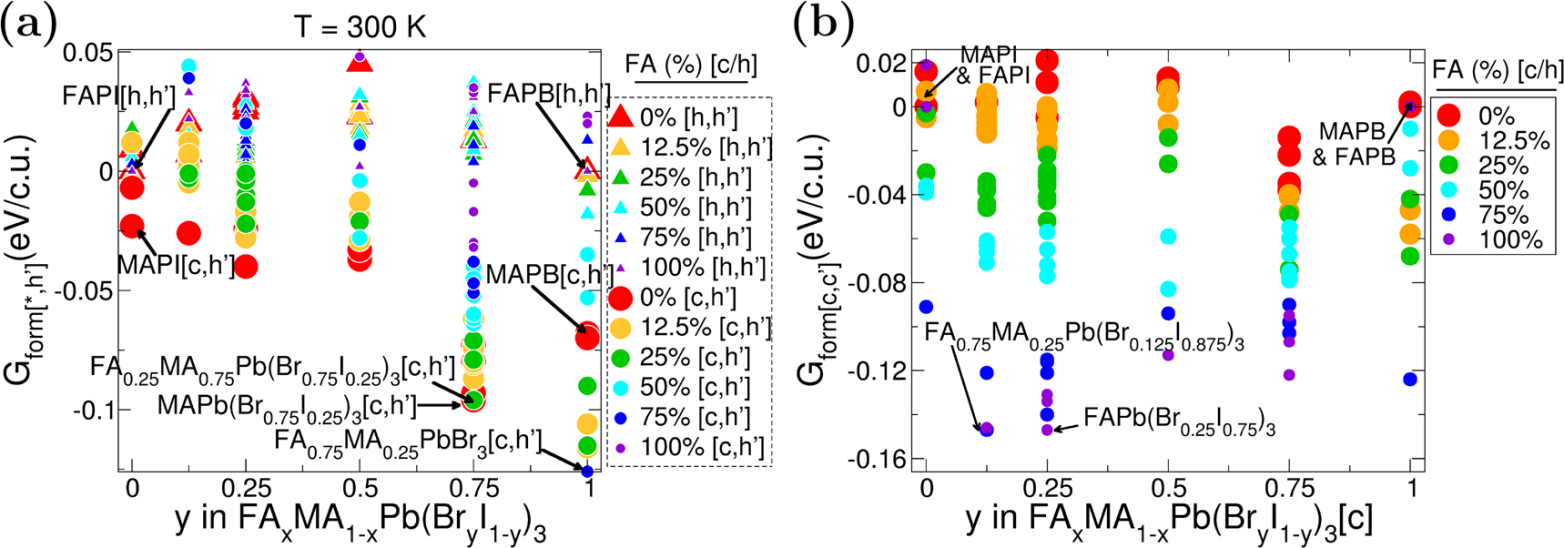
(a) Formation energy of hybrid perovskite with respect to hexagonal constituents and (b) formation energy of hybrid perovskite with respect to same phase constituents at room temperature, both according to eq 5. c.u. stands for chemical unit.

The formation energy of each structure provides an indication as to the stability of each phase as given by eq 5. To do this, we consider several different-site permutations for each stoichiometry as well as differing molecular orientations. The results in Figure 4a show the cubic-like phase is generally more stable than the hexagonal for most stoichiometries considered. This is a consequence of including the room temperature entropy term in eq 4. If one does not include this term, then, in general, the hexagonal phase is more preferable (see Figure S12). We note that this is in agreement with Thind et al.,^50^ who showed the same behavior for MAPI at 0 K.

The results in Figure 4a show that cubic-like mixed perovskites are mostly more favorable to form at room temperature than the segregated constituents in the hexagonal phase. Interestingly, as shown in Figure 4b for most compositions, the solid solution is more energetically favorable than the individual cubic constituents as well. This supports that mixed systems are more stable than the components and prefer the cubic-like phase. A key reason behind the increased stability in Figure 4b is the difference in vibrational free energy between the phases with *F*_vib_ term in cubic-like phase and hexagonal phase, which is significantly different at room temperature. This means that most of the constituents, except FAPI, are strongly unfavorable to be in the hexagonal phase. An exception to this increase in stability for the cubic-like structures are those mixtures with high FA content (≥50%) and low Br content (≤25%). As cubic-like FAPI is extremely unfavorable (0.26 eV per chemical unit), those mixtures which have a significant proportion of FAPI are generally unfavorable at room temperature.

Perovskites in the hexagonal phase mostly have positive formation energies, meaning that the mixed structure in the hexagonal phase is less favorable to form and would prefer to separate into their constituents. This is due to a combined strain effect from the rotational twist of individual PbX_3_ units in its chains (which is unique to the hexagonal phase) and the substantial volumetric strain from Br–I mixing, observed in both phases. The unevenly distributed volume in the hexagonal supercell leads to the three halides in a plane between two lead atoms being rotated to maximize the spacing between the plane and the neighboring planes of halide atoms. This rotational twist, in turn, restricts two molecules to fit within the spacing of every subunit and raises formation energy with respect to the bulk. Therefore, when mixing into FA_*x*_MA_1–*x*_Pb[Br_*y*_I_1–*y*_]_3_, the Br–I mixture reduces the overall volume while the FA and MA molecules compete for maximizing their spacing within the volumetric and rotational strain. This competition within the smaller volume leads to a higher energy requirement for formation compared to constituents.

At room temperature, MAPI and MAPB show cubic-like structures and FAPI and FAPB show hexagonal structures. As stated previously, mixing these results in cubic-like structures in general, and as the Br content increases, the stability of the cubic-like mixed perovskites increases. The most energetically favorable structure, compared to hexagonal constituents at room temperature is cubic-like and has the overall chemical formula of FA_0.25_MA_0.75_Pb[Br_0.75_I_0.25_]_3_ in Figure 4a. This cubic-like composition has a significant negative formation energy, much lower than the corresponding hexagonal phase cell. This result suggests this cubic-like phase is less likely to change to the hexagonal phase at room temperature.

A second criterion for understanding the stability of mixed perovskites is the comparison of each composition to its constituents in the same phase. Positive energy here would indicate the system would prefer to segregate, whereas negative energy indicates a preference for mixing. Figure 4b shows that most of the cubic-like mixed hybrid perovskites have negative formation energies, meaning that mixing is more preferable than individual constituents. The exceptions are all of the MA-based mixed halide perovskites and some of the 12.5% FA mixed halide perovskites, i.e., MAPb(Br_*y*_I_1–*y*_)_3_ and numerous metastable configurations of FA_0.125_MA_0.875_Pb[Br_*y*_I_1–*y*_]_3_. Those MA-based mixed halide perovskites are not favorable to form because (eq 5) the constituents MAPI and MAPB have strong formation energy due to their elongated and compressed Jahn–Teller distortions, respectively.^66^ As the FA content increases, the formation energy of mixed perovskites decreases because the cohesive energies of FAPI and FAPB are weaker than MAPI and MAPB. This indicates that the inclusion of higher amounts of FA improves the intermixing of the solution. According to eq 5, increasing the FA content increases the ratio of the components with a low cohesive energy (FAPI and FAPB) to those with a high cohesive energy and thus favors the formation of mixed perovskites. On the other hand, as the Br content increases, the energetic cost of forming the cubic-like mixed perovskites increases (with respect to the constituents) because the cohesive energies of MAPB and FAPB are stronger than those of MAPI and FAPI. Our results indicate that, compared to its constituents at room temperature, the most favorable in terms of cohesive energy is FA_0.75_MA_0.25_Pb[Br_0.125_I_0.875_]_3_. This is due to its high content of FAPI distributed in the mixture. Furthermore, by analysis of the geometry of the structure, tilting of PbX_6_ octehedra and shortened lattice parameters suggest a compressed Jahn–Teller distortion. Formation energy is thus decreased, indicating higher stability.

To further refine these results, one can examine which structures lie within the thermal energy range (*NK*_B_*T*) at room temperature of their lowest energy configuration. These structures can be obtained via the data in Figures 3 and 4a and examined for improved stability and electronic properties. We now define our desirable features: (i) the cubic-like composition must be more favorable than the hexagonal and (ii) the transition temperature from hexagonal to cubic-like should be low. These two conditions are required for these systems to act as an optimum solar material.

**Figure 5:**
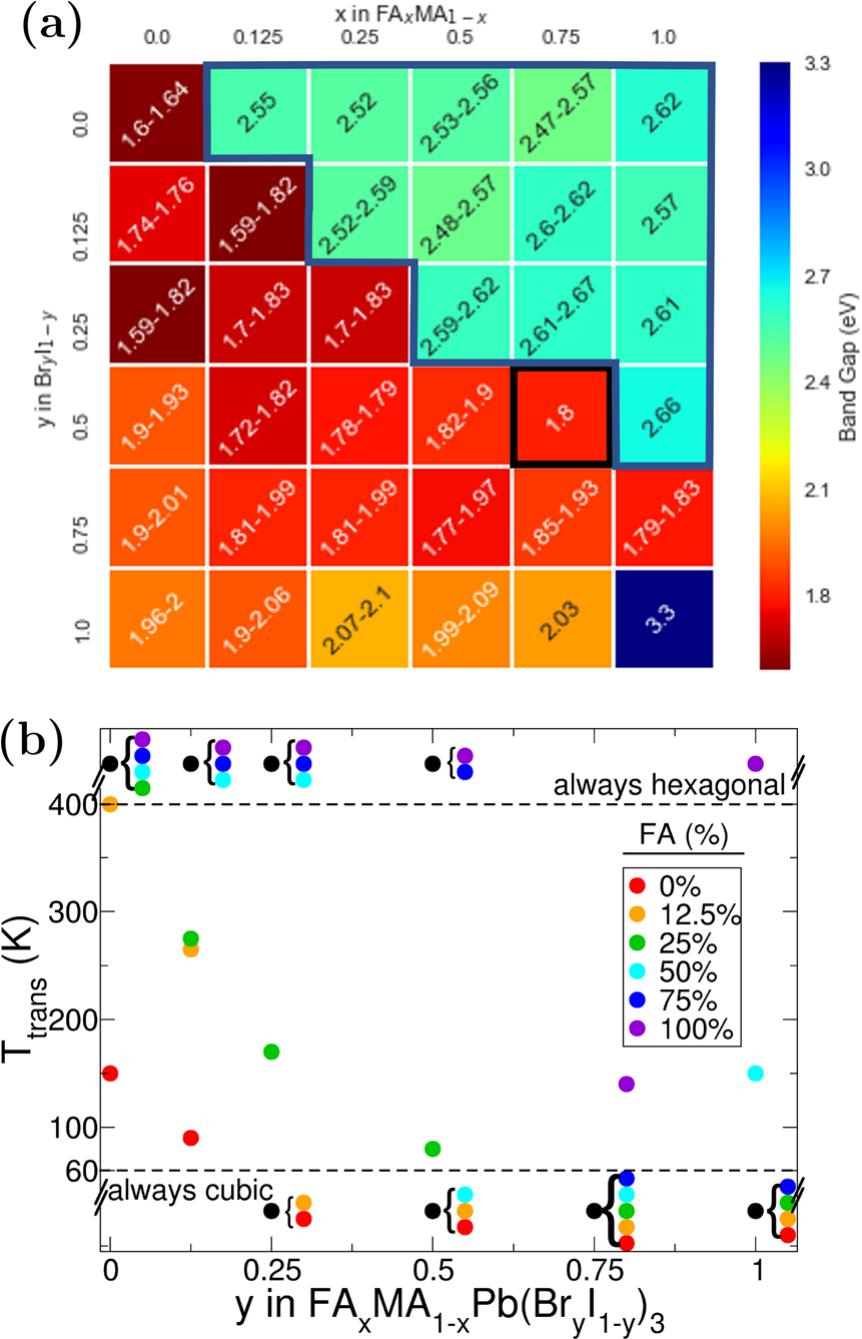
(a) Variation of the band gap with different mixes of FA_*x*_MA_1–*x*_Pb[Br_*y*_I_1–*y*_]_3_ at room temperature. The region with and without the blue box are band gaps of hexagonal and cubic-like structures, respectively. The data with a black box indicates that the band gap of the structure which segregates to constituents. (b) Transition temperature from the hexagonal to cubic-like phase of hybrid perovskite vs composition. For *T*_trans_ ≤ 60 K, perovskites are intrinsically cubic-like. On the other hand, for *T*_trans_ ≥ 400 K, those are intrinsically hexagonal.

Figure 5 shows the electronic band gaps of the stable phases of the various compositions of FA_*x*_MA_1–*x*_Pb[Br_*y*_I_1–*y*_]_3_ at room temperature and their transition temperatures. The results in the figure agree with the general trend shown by Jesper Jacobsson et al.^67^ for the cubic phase. The cubic-like perovskites studied exhibit a band gap from 1.6 eV of MAPI to 2 eV of MAPB. The cubic phases for the low amounts of Br show low gaps of 1.6 eV and below, in agreement with Alsalloum et al.^68^ (see Figure S13a). However, in our calculations, these systems are unstable and will eventually form hexagonal phase perovskites. For systems with *x* > *y*, we expect the rate of decay to the hexagonal phase over time to increase due to the greater the amount of FA compared to Br. The most energetically favorable configuration of FA_0.25_MA_0.75_Pb[Br_0.75_I_0.25_]_3_ exhibits a bandgap of 1.98 eV (unstrained). Thus, we expect that mixed hybrid perovskites will prefer to produce this cubic phase, which is more structurally stable but results in a higher band gap. Strain effects in the solution could change this gap, resulting in some variance.

Similarly, our results in Figure 5b show that within the range of temperature from 60 to 400 K, as *y*, i.e., the Br content, increases, the required transition temperature (from hexagonal to cubic-like phase) decreases. This suggests that the cubic-like phase becomes more favorable than the hexagonal phase with the increasing Br content, hence the increased stability of cubic-like perovskites phases. In addition, as *x*, i.e., the FA content, increases, the transition temperature from the hexagonal to cubic-like phase increases unless higher Br content is included. The figure suggests that for a stable formation of cubic-like perovskites one must not exceed FA content over Br content, i.e., *x* ≤ *y*.

## Conclusion

We have reported the electronic band gaps and stability of FA_*x*_MA_1–*x*_Pb[Br_*y*_I_1–*y*_]_3_ hybrid perovskites. We have shown that the band gap can be heavily influenced by varying the halide composition but is relatively unaffected by choice of either methylammonium or formamidinium. Our study validated that on a more thorough mixing between MA and FA molecules and between Br and I using a first-principles study. Our results also indicate that mixing in a small amount of formamidinium improves the cubic-like phase stability while having a minimal effect on the electronic band gap. In particular, our results indicate that for a stable formation of cubic-like perovskites one must not exceed formamidinium content over Br content, i.e., *x* ≤ *y*. Furthermore, for those solutions with *x* > *y*, the stability of the cubic-like phase can be expected to decrease (and FAPI is the least cubic-like stable phase). This means that to make solar cells with Shockley–Queisser efficiencies of 27.5% or higher, the ratio of I to Br should be higher, and the amount of formamidinium should be kept low. These compositions include FA_*x*_MA_1–*x*_Pb[Br_*y*_I_1–*y*_]_3_ with 0.125 ≤ *x* ≤ 0.25 and 0.125 ≤ *y* ≤ 0.5. For tandem cells, high amounts of Br and formamidinium need to be mixed to make the solution prefer the higher band gap hexagonal phase at room temperature. These results provide promising avenues to refine the development of hybrid organic–inorganic perovskite solar cells and suggest a route to improved stability.

## Supporting Information Available

The Supporting Information contains The Supporting Information is available free of charge at https://pubs.acs.org/doi/10.1021/acs.jpcc.2c03555.

Further details on calculations, including the phonon and entropy calculations; comparison of our results with known results for the bulk phases of MAPI and FAPI and discussion of the effects of molecular orientation of the MA and FA molecules with our results (PDF)
