# Supplementary material for: Phase Stability and Electronic Properties of Hybrid Organic–Inorganic Perovskite Solid Solution (CH(NH2)2)x(CH3NH3)1–xPb(BryI1–y)3 as a Function of Composition
Source: J Phys Chem C Nanomater Interfaces. 2022 Aug 4;126(32):13640–8. doi: 10.1021/acs.jpcc.2c03555 (PMC9393887; doi:10.1021/acs.jpcc.2c03555)
Supplement: Supplementary file 1 — jp2c03555_si_001.pdf [file jp2c03555_si_001.pdf]

Phase Stability and Electronic Properties of Hybrid Organic-Inorganic Perovskite Solid Solution $(CH(NH_2)_2)_x(CH_3NH_3)_{1-x}Pb(Br_yI_{1-y})_3$ as a Function of Composition - Supplementary information.

T. H. Chan,[†] N. T. Taylor,[†] S. Sundaram,[‡] and S. P. Hepplestone[*,†]

†*Department of Physics and Astronomy, College of Engineering, Mathematics and Physical Sciences, Streatham Campus, University of Exeter, UK., EX4 4QL*

‡*Environment and Sustainability Institute, College of Engineering, Mathematics and Physical Sciences, Penryn Campus, University of Exeter, UK., TR10 9FE*

E-mail: S.P.Hepplestone@exeter.ac.uk

Orientation of organic molecule in solid solution supercells

To represent the different orientations potentially possible of the organic molecules (methylammonium or formamidinium), two representative sets of orientations were used as described in the Tables S1 and S2. For the bulk MAPI (and also FAPI) systems, we considered further molecular orientations, by considering 45° (60°) rotational steps for the cubic (hexagonal) phases, and relaxing all these combinations. From these, we chose the two sets below for the solid solution based on these presenting the greatest range of results.

Table S1: Orientations of molecules in cubic perovskite with respect to the first lattice vector.

Molecule no.	$\theta(°)$	$\psi(°)$	$\phi(°)$
1	0	225	135
2	0	315	135
3	0	135	45
4	0	45	45
5	0	45	45
6	0	135	45
7	0	315	135
8	0	225	135

Table S2: Orientations of molecules in hexagonal perovskite with respect to the first lattice vector.

Molecule no.	$\theta(°)$	$\psi(°)$	$\phi(°)$
1	90	0	0
2	90	0	180
3	90	0	180
4	90	0	0
5	90	0	120
6	90	0	300
7	90	0	240
8	90	0	60

Bulk Constituent Properties

Table S3: Lattice parameter of bulk cubic hybrid perovskite.

Hybrid Perovskite	a [Å]
MAPI	6.28[1]
MAPB	5.93[2]
FAPI	6.362[3]
FAPB	5.995[4]

The crystal structure of the hybrid perovskites were cubic, as indicated in References noted in Table S3 and thus all the interaxial angles are at 90°. Our lattices were generated using the replicated lattice parameters.[1–4] To generate the basis within the crystal, we chose to have the centre of the molecule as the origin of the crystal structure because the atomic positions of species in the molecule can be calculated easily by its symmetry. Besides the molecule, the position of the B and X sites is determined in the following table.

Table S4: Crystal coordinates of the inorganic components of hybrid perovskites. X = {Br, I}

Atom Index	x	y	z
Pb	0.500000	0.500000	0.500000
X1	0.500000	0.000000	0.000000
X2	0.000000	0.500000	0.000000
X3	0.000000	0.000000	0.500000

Table S4 shows the atomic position of the inorganic components in direct (crystal) coordinates as they would vary in the form of Cartesian coordinates based on the size of the crystal lattice.

Tables S5 and S6 show the basis of C, H and N in Cartesian coordinates as they would vary in the form of direct coordinates based on the crystal lattice.

Properties in Different Phases

$$\Delta G = G[p] - G[l], \tag{1}$$

Table S5: Cartesian coordinates of MA molecule

Atom Index	x	y	z
C	0.730000	0.000000	0.000000
H1	1.107202	0.921821	-0.533728
H2	1.107202	0.000000	1.065185
N	-0.730000	0.000000	0.000000
H3	-1.063807	-0.815770	0.472326
H4	-1.063807	0.815770	0.472326
H5	-1.063807	0.000000	-0.942641
H6	1.107202	-0.921821	-0.533728

Table S6: Cartesian coordinates of FA molecule

Atom Index	x	y	z
C	0.000000	0.000000	0.000000
H1	1.130000	0.000000	0.000000
N1	-0.730000	0.000000	-1.264397
N2	-0.730000	0.000000	1.264397
H2	-0.230000	0.000000	2.130422
H3	-1.730000	0.000000	1.264397
H4	-0.230000	0.000000	-2.130423
H5	-1.730000	0.000000	-1.264397

where ΔG is the change in Gibbs free energy. [p/l] indicates cubic-like phase or lowest energy phase of all, respectively.

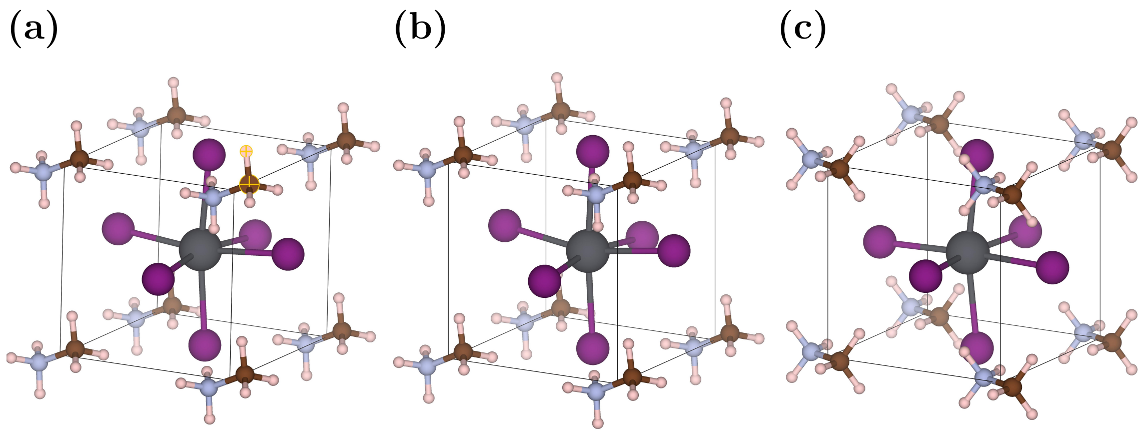

Figure S1: Fully geometrically optimised structure of MAPI in (a) alongside with partially optimised (b) tetragonal and (c) cubic phases.

The three optimised cubic-like structures of MAPI in various phases are shown in Figure S1. The orthorhombic structure has uneven lattice parameters, varying from 6.276 to 6.395 $\mathring{A}$. The tetragonal structure was set to 6.248 $\mathring{A}$ in a and b and 6.516 $\mathring{A}$ in c. Moreover, the cubic structure was set to 6.333 $\mathring{A}$ in all lattice parameters. Regarding the interaxial angles, all the phases maintain the angles within only 1.5° away from right angles.

When relaxing the tetragonal and cubic structures fully, they return to the orthorhombic phase, showing the orthorhombic phase is more preferable at zero temperature. We calculate the Gibbs free energies (Eqn. (1)) to form MAPI in the tetragonal phase and cubic phase, given that temperature is assumed at 300 K, are 0.009 eV and -0.002 eV respectively. The little difference in Gibbs energy difference suggests that the orthorhombic and cubic phases are energetically favourable to transition to one another. On the other hand, the tetragonal phase is comparably unfavourable.

The band gaps of orthorhombic, tetragonal and cubic are 1.58, 1.52 and 1.59 eV, respectively, in Figure S2, which are in agreement with Baikie *et al.*,[5] except the cubic band gap. The cubic structure has a slightly higher band gap because the result of Baikie *et al.*

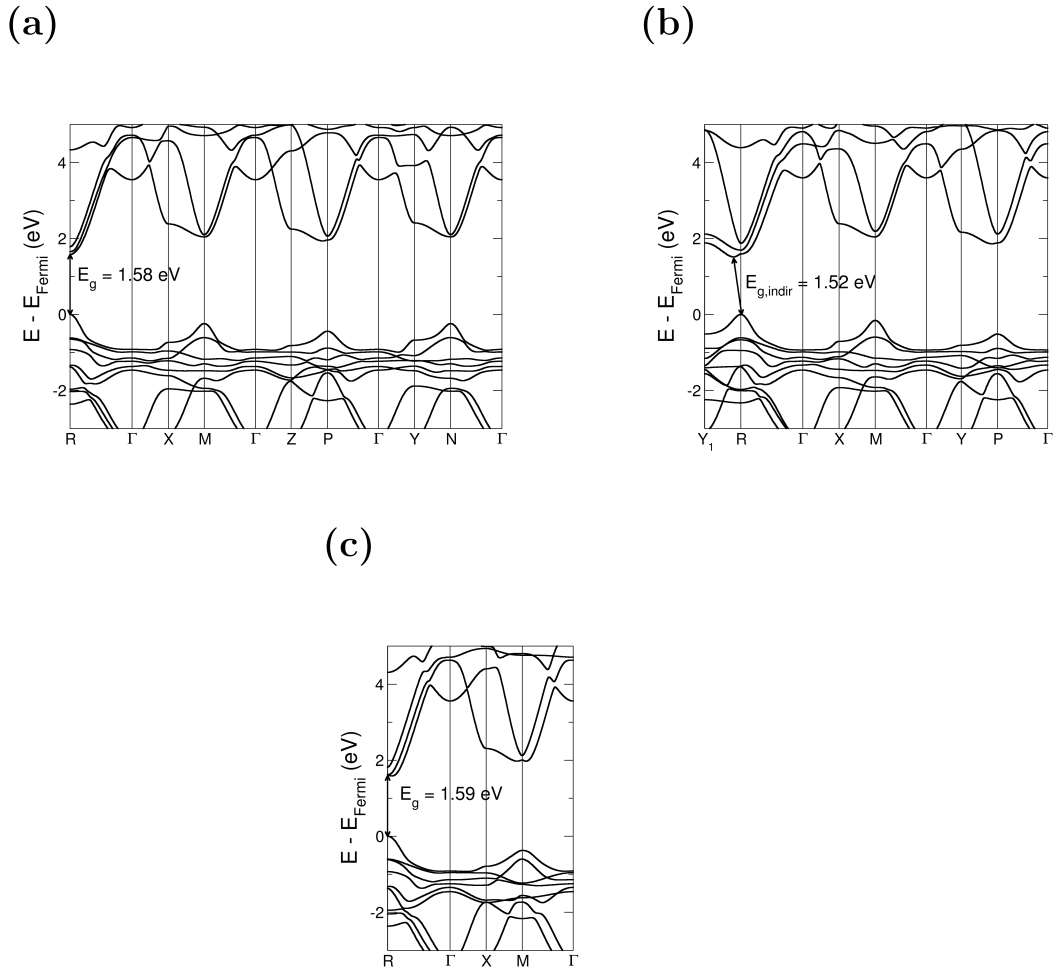

Figure S2: Electronic band structures of MAPI in (a) orthorhombic, (b) tetragonal and (c) cubic phases. The crystal coordinates of the labelled high-symmetry **k**-points are discussed in Table S10.

adopted substitution of MA molecule by a monovalent metal cation to perform structural relaxation and applied a uniform charge density, leading to the underestimation of lattice parameters and thus the band gap. Our calculated gap of cubic MAPI agrees with Kim *et al.*[6]

The orthorhombic and cubic structures share a similar direct band gap of 0.01 eV difference, which is at the same high-symmetry **k**-point. This suggests that the orthorhombic phase should be used for further calculations and the electronic band structure can be reduced as the orthorhombic phase is slightly more preferable than the cubic phase at zero

temperature.

In the case of the tetragonal band structure in Figure S2b, the band gap is 1.52 eV, in agreement with Baikie *et al.*,[5] which is 0.06 eV lower than the gaps in the orthorhombic and cubic phase. This is due to the strain effect of a 4% longer lattice parameter along the vertical axis than that of the cubic phase. This leads to the band splitting near the conduction bands of the tetragonal structure from the degenerate CBM in the cubic structure.[7] Another study by Park *et al.*[8] suggests that the tetragonal MAPI should exhibit as high as 1.6 eV but the use of $PbCl_2$ indicates the composition should be mixed I-Cl with an unknown concentration of chlorine present that could lead to the increase in the band gap.

In summary, our calculations have shown that the band gaps vary by only 0.07 eV. The orthorhombic phase of MAPI exhibits the lowest band gap and its formation energy has shown to be the most favourable among the cuboid phases. For future investigation, the orthorhombic phase of hybrid perovskite will be taken into account.

Rotation Energy

The energy between different molecular orientations (rotational configurations) E_{rot} is given by

$$E_{rot} = \frac{E_{tot} - E_{aligned}}{n} \tag{2}$$

where E_{rot} is the energy difference between the rotated configuration with respect to the aligned molecules. For simplicity, we term this as rotational energy. E_{tot} is the total energy of the rotated-molecule structure, $E_{aligned}$ is the total energy of the system with aligned molecules for reference, and n is the number of unit cells studied.

Molecular Rotation of Metastable Frozen Structure

Here, we look into the effect of rotating a molecule within a geometrically optimised system that has aligned molecules. The rotation energies required to rotate a pre-aligned molecule in a geometrically optimised MAPI is shown in Figure S3a. The angle of rotation is with respect to the aligned orientation of the molecules. By comparing the rotation along the three axes, the energetic cost to rotate around ϕ and ψ are similar and higher than around θ due to the deformation of PbI_6 cage around the MA molecule.[10] The rotation energy required for the full rotational profile could go as high as 0.32 eV/(u.c.) in Figure S3a (0.15 eV/(u.c.) for FA molecule in Figure S4a) which will be highly unlikely, given that the thermal excitation energy at room temperature is about 0.03eV/(u.c.), as illustrated with the dotted line. This result of rotational energy agrees with the study of Quarti *et al.* which found the apolar orientation of MA molecules requires 0.2 eV/(u.c.)[11] Another interesting feature is that several saddle points of rotation energy are located near 90°, 180° and 270° from the neighbouring MA molecule in both rotations around the axes parallel and in-plane perpendicular to C-N bonds, i.e. θ and ϕ. However, these saddle points are associated with high rotation energy. On the other hand, for the FA molecule in Figure S4a, most of the saddle points (45°, 135°, 225° and 315°) are within the thermal excitation energy at room temperature. These suggest the metastable states could be present which predicts to decrease the band gap of perovskite by 0.05 eV in Figure S4b.

The band gap associated with the rotation of the MA molecule is shown in Figure S3b. The grey portion of the curve indicates such band gaps are unfavourable as such configuration requires more energy than the thermal excitation at room temperature. In the very unlikely event, the MA (FA) molecules could flip by 180° in the perovskite in any direction that leads to up to a 0.4 (0.05) eV decrease under isochoric conditions. By combining the discussions of the result of energetics and the electronic band gap, we could expect that some permutations are unfavourable to form even with the aid of thermal excitation. Thereby, the MA molecular rotation should be limited to 90° in θ, 30° in ϕ and ψ. Within this range of rotation, we expect the band gap of the hybrid perovskite fluctuates within $\pm$ 0.05 eV. Specifically, MAPI

is calculated to have a band gap of 1.55 to 1.6 eV which is in agreement with the studies by Kojima, Stoumpos *et al.*[12,13] However, for molecules to rotate within the hybrid perovskite, the local charge density is expected to vary and this could impact the external potential to neighbouring atoms. Thus, to resolve the full effect of molecular rotation to energetics and electronic properties of the system, structural relaxation should be further considered to allow atoms to displace to an equilibrium position and thus the volume of the hybrid perovskite is expected to change and subsequently the band gap of the system.

In a summary of the results of molecular rotation in hybrid perovskites at a metastable configuration, they suggest that the measured band gap of the same sample can fluctuate by 0.05 eV at room temperature regardless of the pristine bulk perovskite used.

Metastable Rotational Configuration

Here, we investigate the potential metastable states of the bulk hybrid perovskites. To achieve these, the $2 \times 1 \times 1$ supercell of MAPI and FAPB are computed. One of their molecules is rotated prior to structural optimisation. The range of orientations θ, ϕ and ψ considered is reduced to 0° to 180° with an increment of 45° as the fluctuation of rotation from the originally aligned molecules in Section showed a similar trend of rotation energy and electronic band gap when molecule rotating clockwise or anti-clockwise. Subsequently, electronic relaxation was performed. The energy difference E_{rot} was compared among the different configurations. This investigation is to analyse various potential metastable phases to coexist in the same condition.

The energy difference between the structurally optimised hybrid perovskites with various molecular orientations and their corresponding electronic band gaps are shown in Figures S5 and S6. This study has shown significance as the structurally optimised states could be metastable. Figure S5 indicates that perovskites with MA molecules can have many metastable configurations where molecules had various orientations within the thermal excitation at room temperature. From that, we can expect variations of band gap up to 0.1

eV (0.3 eV) in MAPI (MAPB). On the other hand, according to Figure S6, FA molecules in FAPI and FAPB are constrained to a specific orientation. Even with thermal excitation energy at room temperature, it is not sufficient for molecules to freely rotate and form a metastable configuration of the entire bulk system. The range of band gap of FAPB due to rotation agrees with the study by Al-Kahtani *et al.*[14] Thus, the band gap of FAPI and FAPB remain at 1.44 eV and 1.85 eV.

In summary of studying the metastable rotational configuration, we can expect between samples of MAPI and between those of MAPB, there could be random orientations of MA molecules within the sample that exhibit band gaps up to 0.1 and 0.3 eV difference, respectively. However, the measured band gap should remain the lowest of them, which are 1.58 eV and 1.75 eV, respectively. For FAPI and FAPB, the band gaps between the sample should be consistently 1.44 eV and 1.85 eV.

Vibrational Free Energy

Table S7: Zero-point vibrational energy ϵ of hexagonal/cubic-like $[c/h]$ hybrid perovskite in eV.

Hybrid Perovskite	$\epsilon[c](eV)$	$\epsilon[h](eV)$
MAPI	34.1477	34.1954
MAPB	34.4853	34.5114
FAPI	30.9853	31.3532
FAPB	31.5903	31.7008

Figures S7 and S8 presents the correlation between vibrational entropy S and temperature T. Its unit is [$JK^{-1}mol^{-1}$] in the table so it is in line with the result shown in the paper of Butler *et al.*[15] However, for the calculation in Equation 4, S was converted into S_{vib} in eV/K after multiplying by the Avogadro's number and dividing by 1.602 $\times 10^{-19}$.

The tetragonal and cubic structures were resulted from constraining the optimised cubic-like structure and they were all calculated in PHONOPY. After data analysis, they show that the cubic-like optimised structure and the cubic structure have almost identical entropy

across the range of temperatures and their entropies are higher than the entropies of the tetragonal structure. This reproduces the same trend as shown in the result from Butler *et al.*. The magnitude of the entropy from our result is different as our structures are 2 × 2 × 2 supercells whereas the MAPI structure from the paper was unidentified.

Figure S9 shows the phonon band structure of cubic-like MAPI. The bottom three bands are the acoustic modes which atoms are vibrating in-phase to each other in the three primary directions. They mostly show negative phonon frequencies along the high-symmetry k path which suggests the MAPI structure is unstable. This is because the use of PBE functional overestimates the optimisation of lattice parameters. A similar study of the cubic-like MAPI supercell using PBEsol functional by Brivio *et al.*[16] displayed negative phonon frequencies at acoustic modes near some of the non-Γ high-symmetry k-points. They represented the antiferroelectric distortions related to the rotations and tilting of inorganic octahedra.

By comparing the calculated phonon band structure with the literature, we notice softening in phonon frequencies in our cubic-like unit cell, especially at Γ point. This is due to 2% lower in the volume of the cubic-like unit cell using PBE functional as opposed to that using PBEsol in the literature by Brivio *et al.*

For more accurate phonon frequencies and band structure, a larger supercell should have been used, but within our study, the computational cost would be unfeasible.

Validity of the use of functionals

In the main results, we showed that the use of the PBE functional and D3 vdW correction in DFT calculations resulted in an agreeable electronic band gap of hybrid perovskites to that observed in experimental studies. To further justify the adoption, we tested the structurally optimised structures of a single chemical unit of MAPI and FAPB using hybrid functional HSE06 or spin-orbit coupling (SOC) or both. These were Γ point only calculations, only probing the band structure at the centre of the Brillouin zone due to computational cost.

Table S8 records the total energy and the electronic band gap calculated from DFT using various exchange-correlation functionals and corrections. As can be seen, the gains for examining the electronic gap for these mixed structures, compared to the PBE GGA functional, are relatively small. However, for the scale and number of structures considered in the main article, the HSE functional is prohibitively computationally expensive for little gain in terms of insight. For investigating purely the optical gap (ignoring spin effects) for a high number of structures consider, we have used PBE without SOC due to the closeness to the experiment and due to its computational feasibility.

Table S8: Electronic band gaps E_g of MAPI and FAPB calculated from various functionals and corrections. HSE stands for HSE06 specifically.

	PBE	(+D3)	HSE	(+D3)
MAPI	1.78	1.58	2.29	2.16
FAPB	2.00	1.86	2.53	2.53
	PBE+SOC	(+D3)	HSE+SOC	exp
MAPI	0.55	0.60	1.10	1.59[17]
FAPB	0.90	0.90	3.42	2.23[18]

Table S8 shows that the MAPI band gap calculated using PBE functional and D3 VdW correction agrees with the experimental band gap, but without the D3 correction, the band gap is overestimated. This is due to the overestimation of exchange energy in the exchange-correlation term. For FAPB, the closest band gap to the experimental value is calculated in PBE without D3 vdw correction (0.3 eV below) but in order to keep all calculations comparable, PBE + D3 is used. Our calculated band gaps including SOC and HSE agreed with other publications as well.[19,20] Alternatively, using the HSE06 hybrid functional and SOC correction can also result in a lower band gap of MAPI than experimental by cancelling the error, agreed with Sun *et al.*[21] Furthermore, including both corrections for FAPB shows a clear overestimation compared to the experiment but the computational cost is extremely high, which makes the calculation unfeasible.

Variation of the band gap with changing ratio of methylammonium to formamidinium

As discussed in the main text, there is little correlation between the ratio of methylammonium to formamidinium and either the volume, or the bandgap, as shown in Figure S10. The reason for this lack of correlation is that when the system relaxes, the organic molecule compensates for the strain created by the choice of I or Br, and the local structure.

Bader Charge Analysis

Atom Index	Bader Charge [c]	Bader Charge [h]
Pb1	2.893	2.926
Pb2	2.885	2.917
Pb3	2.886	2.919
Pb4	2.892	2.920
Pb5	2.890	2.917
Pb6	2.889	2.918
Pb7	2.889	2.922
Pb8	2.890	2.921
I1	7.626	7.591
I2	7.619	7.607
I3	7.609	7.592
I4	7.601	7.617
I5	7.601	7.597
I6	7.609	7.619
I7	7.619	7.605
I8	7.626	7.609
I9	7.607	7.605
I10	7.619	7.611
I11	7.619	7.597
I12	7.607	7.608
I13	7.610	7.605
I14	7.611	7.609
I15	7.612	7.598
I16	7.610	7.626
I17	7.621	7.602
I18	7.609	7.595
I19	7.609	7.591
I20	7.621	7.618
I21	7.612	7.601
I22	7.611	7.610
I23	7.611	7.607
I24	7.612	7.602

Atom Index	Bader Charge [c]	Bader Charge [h]
C1	3.503	3.535
C2	3.500	3.502
C3	3.496	3.495
C4	3.530	3.477
C5	3.468	3.507
C6	3.487	3.551
C7	3.503	3.510
C8	3.484	3.509
H1	0.000	0.939
H2	0.923	0.924
H3	0.950	0.000
H4	0.938	0.000
H5	0.000	0.000
H6	0.000	0.929
H7	0.000	0.947
H8	0.943	0.942
H9	0.942	0.000
H10	0.941	0.000
H11	0.000	0.000
H12	0.000	0.934
H13	0.000	0.943
H14	0.934	0.931
H15	0.957	0.000
H16	0.926	0.000
H17	0.000	0.000
H18	0.000	0.948
H19	0.000	0.953
H20	0.941	0.960
H21	0.951	0.000
H22	0.907	0.000
H23	0.000	0.000
H24	0.000	0.940

The Bader charge of Pb and I atoms in hexagonal and cubic-like MAPI is shown in Table S9. By summing the net charges of C, H and N, each MA molecule equips $0.73+$ |e|. Each Pb has a net charge of $1.11+$ |e| whereas each I has a net charge of $0.61-$ |e|. Between the hexagonal and cubic-like structures, the Bader net charge of individual atoms varies up to $0.03 \pm$ |e|. The overall charges cancel out.

Crystal Coordinate of High Symmetry k-points

Band Gaps of Mixed Perovskites presented by phase

Figure S13a shows the bandgaps of the two separate phases of hexagonal and cubic as a function of composition. We note that the lowest bandgaps (ignoring FAPI) are observed for the 50% mix of methylammonium and formamidinium and 100% iodine. However, our results indicate that this phase is ultimately unstable and will decay into the hexagonal phase.

Polyhedra tilt data

To further analyse the data used throughout this manuscript, we also examined the role of the tilting of the polyhedra in each of our supercells. We present these results in Figure S14a. We find that the tilt is generally reduced with increasing formamidinium, but this does not correlate with the bandgap, which is more determined by the Br:I ratio as discussed in the main manuscript.

Table S9: Bader charge analysis of a MAPI in cubic-like and hexagonal phases.

Atom Index	Bader Charge [c]	Bader Charge [h]
H25	0.000	0.921
H26	0.951	0.966
H27	0.946	0.000
H28	0.960	0.000
H29	0.000	0.000
H30	0.000	0.934
H31	0.000	0.919
H32	0.935	0.921
H33	0.960	0.000
H34	0.931	0.000
H35	0.000	0.000
H36	0.000	0.937
H37	0.000	0.954
H38	0.937	0.933
H39	0.943	0.000
H40	0.943	0.000
H41	0.000	0.000
H42	0.000	0.927
H43	0.000	0.938
H44	0.940	0.939
H45	0.949	0.000
H46	0.939	0.000
H47	0.000	0.000
H48	0.000	0.942
N1	7.948	7.940
N2	7.953	7.937
N3	7.959	7.943
N4	7.943	7.937
N5	7.947	7.941
N6	7.958	7.938
N7	7.953	7.936
N8	7.950	7.937

Table S10: High symmetry k-points used to construct the k-path in the band structure in Figures S2a, S2b and S2c

**k**-point	$\mathbf{b}_1$	$\mathbf{b}_2$	$\mathbf{b}_3$
Γ	0	0	0
X	0.5	0	0
Y	0	0.5	0
Z	0	0	0.5
M	0.5	0.5	0
N	0	0.5	0.5
P	0.5	0	0.5
R	0.5	0.5	0.5
Y_1	0	0.5	0.5

References

(1) Frost, J. M.; Butler, K. T.; Brivio, F.; Hendon, C. H.; van Schilfgaarde, M.; Walsh, A. Atomistic origins of high-performance in hybrid halide perovskite solar cells. *Nano letters* **2014**, *14*, 2584–90.

(2) Lehmann, F.; Franz, A.; Többens, D. M.; Levcenco, S.; Unold, T.; Taubert, A.; Schorr, S. The phase diagram of a mixed halide (Br, I) hybrid perovskite obtained by synchrotron X-ray diffraction. *RSC Advances* **2019**, *9*, 11151–11159.

(3) Weller, M. T.; Weber, O. J.; Frost, J. M.; Walsh, A. Cubic Perovskite Structure of Black Formamidinium Lead Iodide, α-[HC(NH2)2]PbI_3, at 298 K. *Journal of Physical Chemistry Letters* **2015**, *6*, 3209–3212.

(4) Pisanu, A.; Patrini, M.; Malavasi, L.; Mosconi, E.; Mahata, A.; Quadrelli, P.; De Angelis, F.; Milanese, C. Exploring the Limits of Three-Dimensional Perovskites: The Case of FAPb 1– x Sn x Br 3. *ACS Energy Letters* **2018**, *3*, 1353–1359.

(5) Baikie, T.; Fang, Y.; Kadro, J. M.; Schreyer, M.; Wei, F.; Mhaisalkar, S. G.; Graetzel, M.; White, T. J. Synthesis and crystal chemistry of the hybrid perovskite

$(CH_3NH_3)PbI_3$ for solid-state sensitised solar cell applications. *Journal of Materials Chemistry A* **2013**, *1*, 5628.

(6) Kim, J.; Lee, S. C.; Lee, S. H.; Hong, K. H. Importance of orbital interactions in determining electronic band structures of organo-lead iodide. *Journal of Physical Chemistry C* **2015**, *119*, 4627–4634.

(7) Even, J.; Pedesseau, L.; Jancu, J. M.; Katan, C. Importance of spin-orbit coupling in hybrid organic/inorganic perovskites for photovoltaic applications. *Journal of Physical Chemistry Letters* **2013**, *4*, 2999–3005.

(8) Park, J. H.; Seo, J.; Park, S.; Shin, S. S.; Kim, Y. C.; Jeon, N. J.; Shin, H. W.; Ahn, T. K.; Noh, J. H.; Yoon, S. C. et al. Efficient $CH_3NH_3PbI_3$ Perovskite Solar Cells Employing Nanostructured p-Type NiO Electrode Formed by a Pulsed Laser Deposition. *Advanced Materials* **2015**, *27*, 4013–4019.

(9) Stamplecoskie, K. G.; Manser, J. S.; Kamat, P. V. Dual nature of the excited state in organic-inorganic lead halide perovskites. *Energy and Environmental Science* **2015**, *8*, 208–215.

(10) Motta, C.; El-Mellouhi, F.; Kais, S.; Tabet, N.; Alharbi, F.; Sanvito, S. Revealing the role of organic cations in hybrid halide perovskite $CH_3NH_3PbI_3$. *Nature Communications* **2015**, *6*, 7026.

(11) Quarti, C.; Mosconi, E.; De Angelis, F. Interplay of orientational order and electronic structure in methylammonium lead iodide: Implications for solar cell operation. *Chemistry of Materials* **2014**, *26*, 6557–6569.

(12) Kojima, A.; Teshima, K.; Shirai, Y.; Miyasaka, T. Organometal halide perovskites as visible-light sensitizers for photovoltaic cells. *Journal of the American Chemical Society* **2009**, *131*, 6050–6051.

(13) Stoumpos, C. C.; Malliakas, C. D.; Kanatzidis, M. G. Semiconducting tin and lead iodide perovskites with organic cations: Phase transitions, high mobilities, and near-infrared photoluminescent properties. *Inorganic Chemistry* **2013**, *52*, 9019–9038.

(14) Al-Kahtani, A. A.; Tabassum, S.; Raya, I.; Khlewee, I. H.; Chupradit, S.; Davarpanah, A.; Elveny, M.; Ali, S. Influence of different rotations of organic formamidinium molecule on electronic and optical properties of FAPbBr3 perovskite. *Coatings* **2021**, *11*, 1–15.

(15) Butler, K. T.; Svane, K.; Kieslich, G.; Cheetham, A. K.; Walsh, A. Microscopic origin of entropy-driven polymorphism in hybrid organic-inorganic perovskite materials. *Physical Review B* **2016**, *94*, 180103.

(16) Brivio, F.; Frost, J. M.; Skelton, J. M.; Jackson, A. J.; Weber, O. J.; Weller, M. T.; Goñi, A. R.; Leguy, A. M.; Barnes, P. R.; Walsh, A. Lattice dynamics and vibrational spectra of the orthorhombic, tetragonal, and cubic phases of methylammonium lead iodide. *Physical Review B - Condensed Matter and Materials Physics* **2015**, *92*, 144308.

(17) Jesper Jacobsson, T.; Correa-Baena, J. P.; Pazoki, M.; Saliba, M.; Schenk, K.; Grätzel, M.; Hagfeldt, A. Exploration of the compositional space for mixed lead halogen perovskites for high efficiency solar cells. *Energy and Environmental Science* **2016**, *9*, 1706–1724.

(18) Eperon, G. E.; Stranks, S. D.; Menelaou, C.; Johnston, M. B.; Herz, L. M.; Snaith, H. J. Formamidinium lead trihalide: A broadly tunable perovskite for efficient planar heterojunction solar cells. *Energy and Environmental Science* **2014**, *7*, 982–988.

(19) Pandech, N.; Kongnok, T.; Palakawong, N.; Limpijumnong, S.; Lambrecht, W. R.; Jungthawan, S. Effects of the van der Waals Interactions on Structural and Electronic Properties of CH_3NH_3(Pb,Sn)(I,Br,Cl)3Halide Perovskites. *ACS Omega* **2020**, *5*, 25723–25732.

(20) Ali, R.; Zhu, Z. G.; Yan, Q. B.; Zheng, Q. R.; Su, G.; Laref, A.; Saraj, C. S.; Guo, C. Compositional Engineering Study of Lead-Free Hybrid Perovskites for Solar Cell Applications. *ACS Applied Materials and Interfaces* **2020**, *12*, 49647.

(21) Sun, P. P.; Li, Q. S.; Yang, L. N.; Li, Z. S. Theoretical insights into a potential lead-free hybrid perovskite: Substituting Pb2+ with Ge2+. *Nanoscale* **2016**, *8*, 1503–1512.

(a)

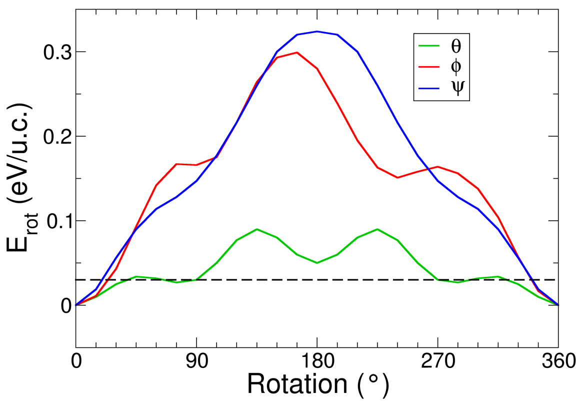

(b)

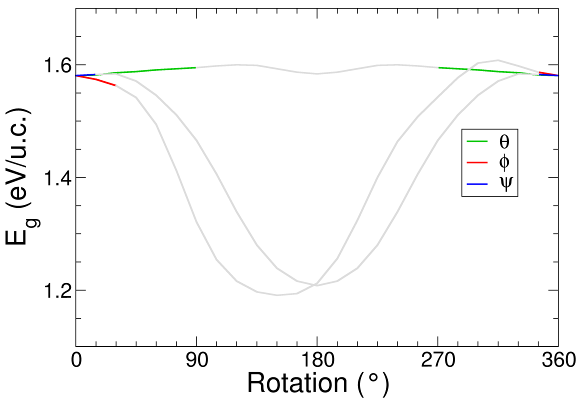

Figure S3: (a) Correlation between rotation energy and angle of molecular rotation in a geometrically optimised MAPI. The dotted line shows the 0.03 eV/(u.c.) from thermal excitation.[9] The corresponding band gap is shown in (b). Grey-scale indicates the energetically unfavourable rotations. It is worth noting that volume of the structure is constant in this calculation.

(a)

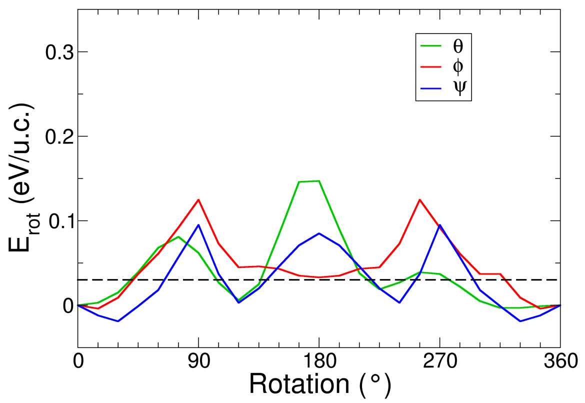

(b)

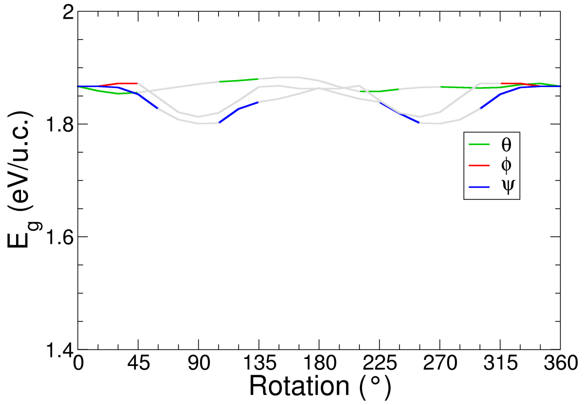

Figure S4: (a) Correlation between rotation energy and angle of molecular rotation in a geometrically optimised FAPB. The dotted line shows the 0.03 eV/(u.c.) from thermal excitation.[9] The corresponding band gap is shown in (b). Grey-scale indicates the energetically unfavourable rotations. Volume of the structure is constant in this calculation.

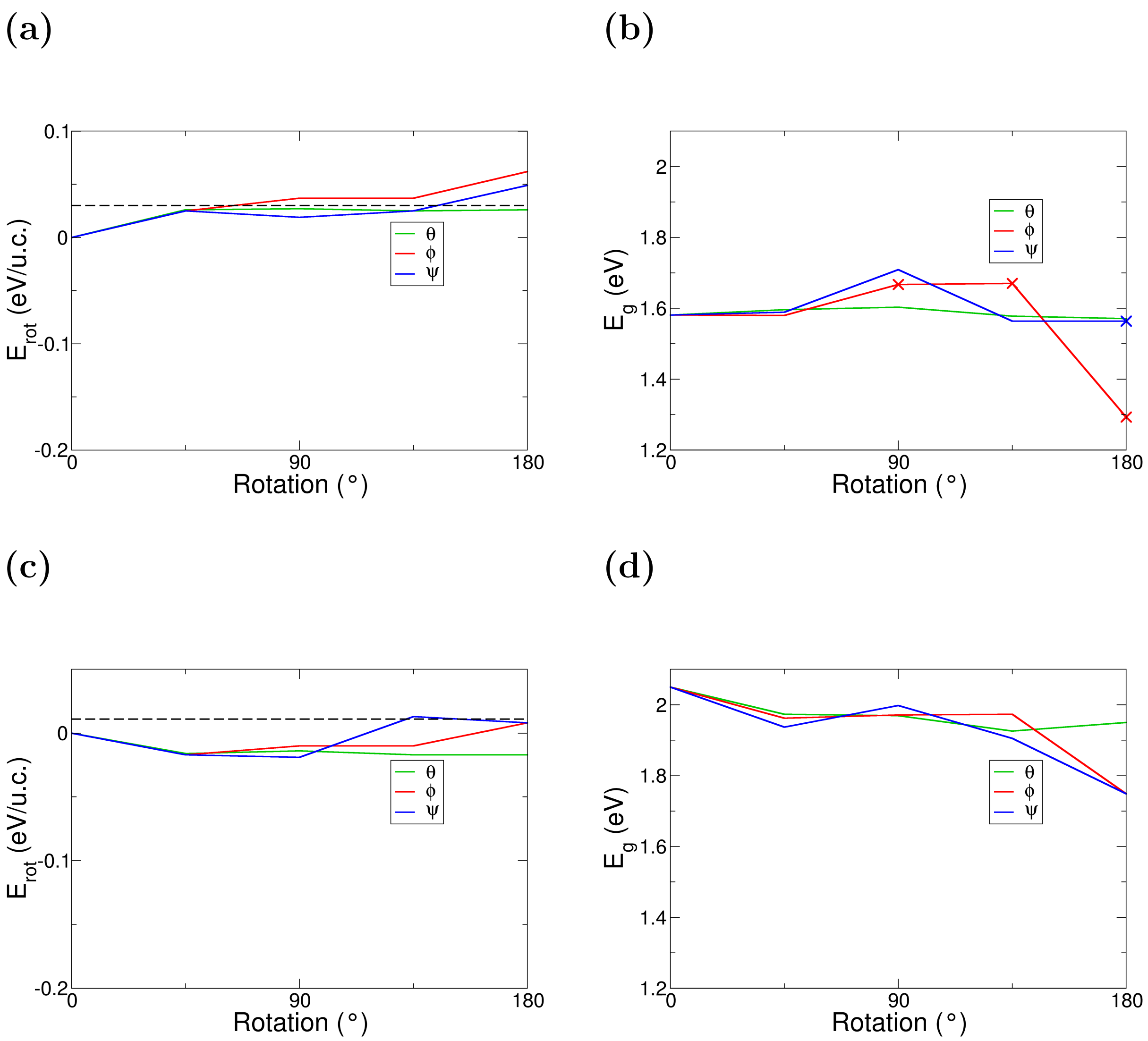

Figure S5: Correlation between rotation energy and angle of molecular rotation between optimised states of (a) MAPI and (c) MAPB. The dotted line shows the 0.03 eV/(u.c.) from thermal excitation.[9] The corresponding band gap of (b) MAPI and (d) MAPB is shown. Data marked with cross indicates the energetically unfavourable rotations.

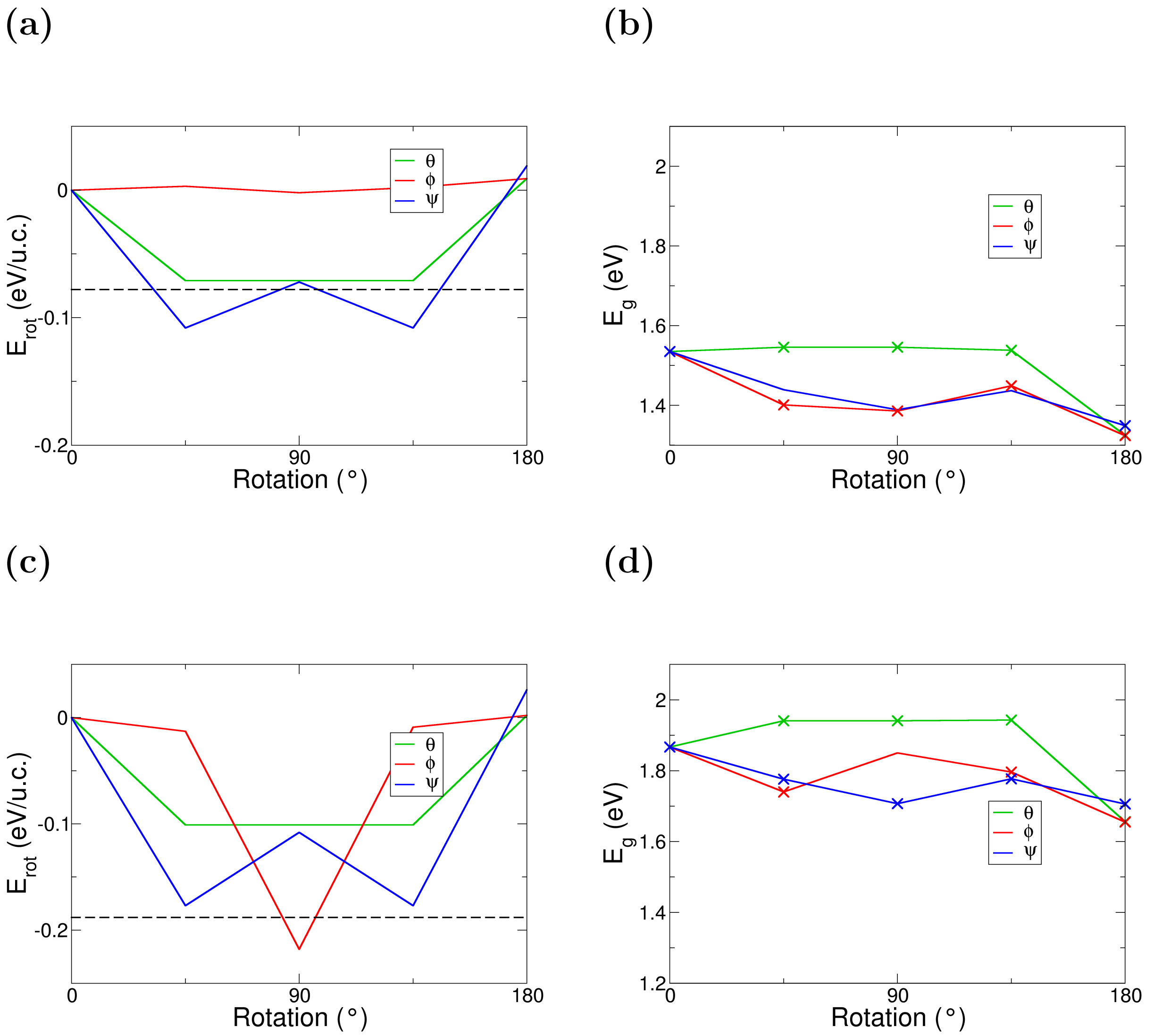

Figure S6: Correlation between rotation energy and angle of molecular rotation between optimised states of (a) FAPI and (c) FAPB. The dotted line shows the 0.03 eV/(u.c.) from thermal excitation.[9] The corresponding band gap of (b) FAPI and (d) FAPB is shown. Data marked with cross indicates the energetically unfavourable rotations.

(a)

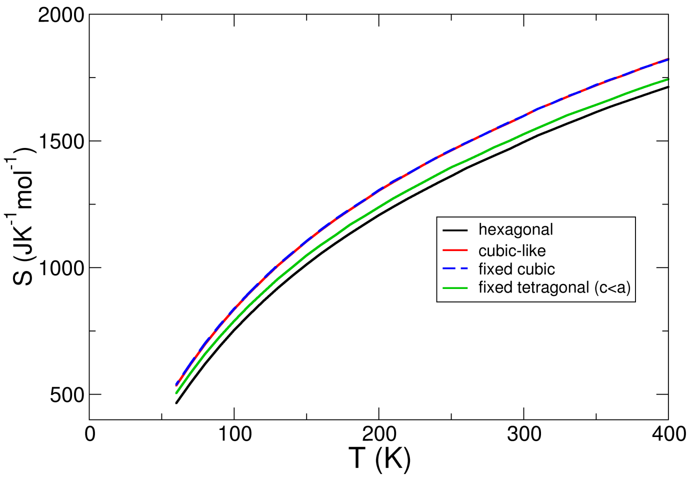

(b)

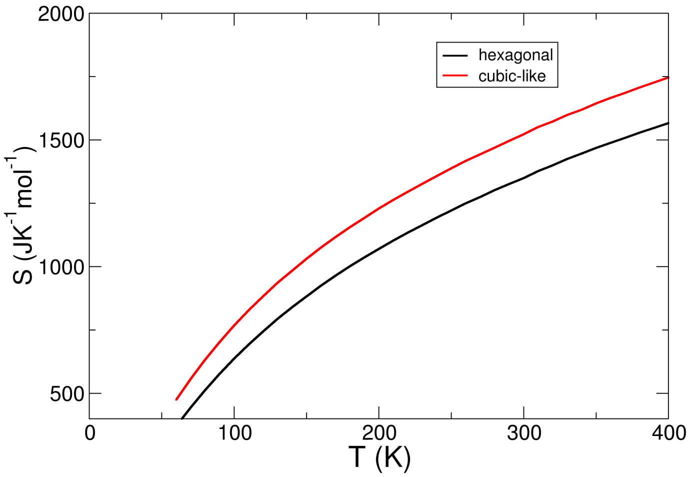

Figure S7: Vibrational Entropy S of (a) MAPI, (b) MAPB, in function of temperature T.

(a)

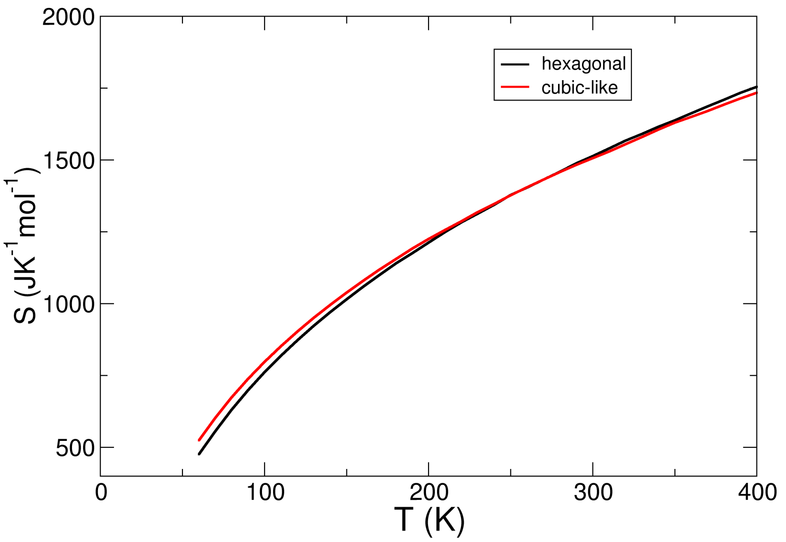

(b)

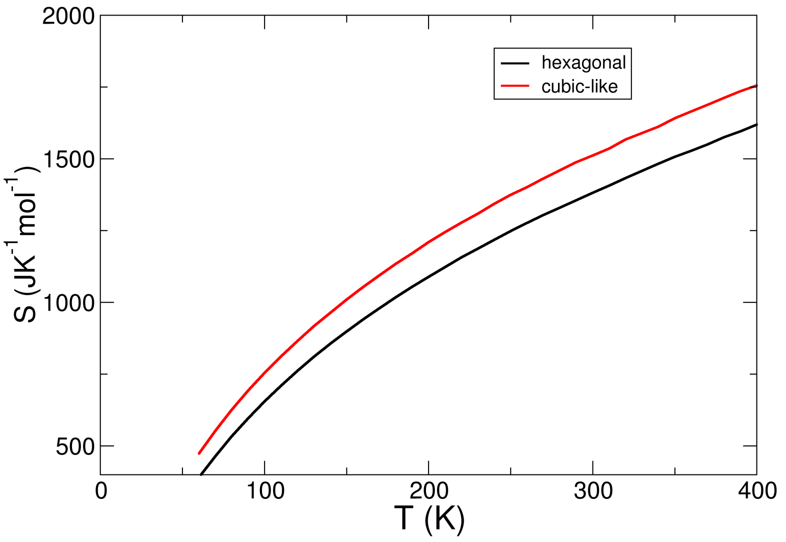

Figure S8: Vibrational Entropy S of (a) FAPI and (b) FAPB in function of temperature T.

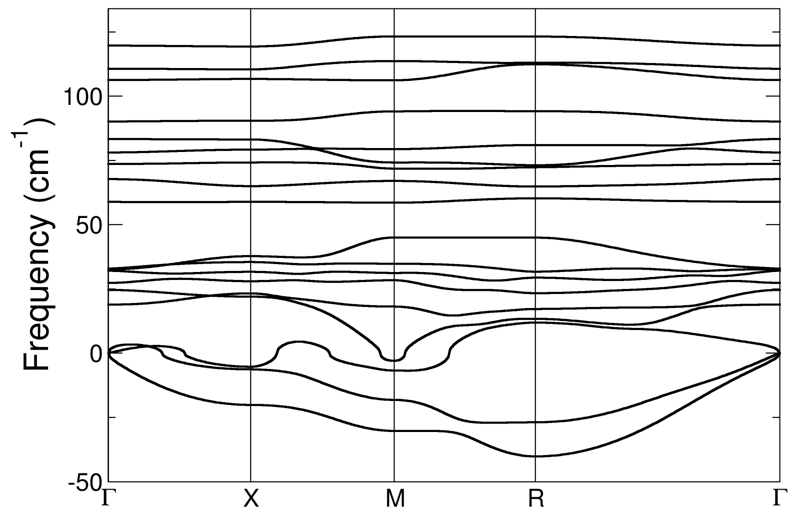

Figure S9: Phonon band structure of cubic-like MAPI

(a)

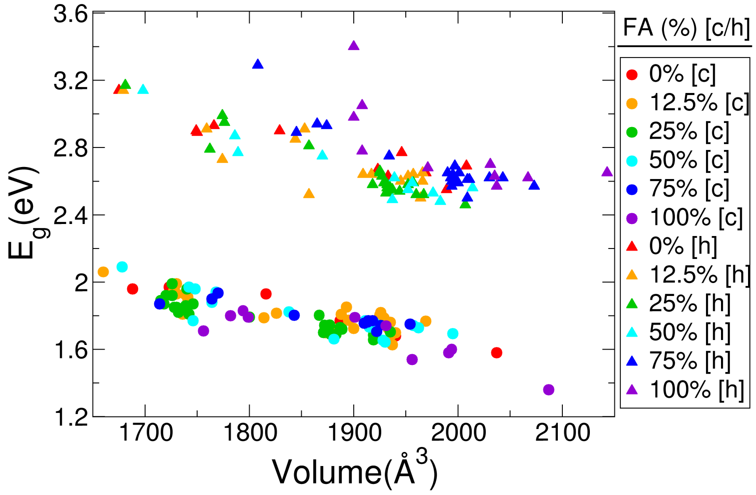

(b)

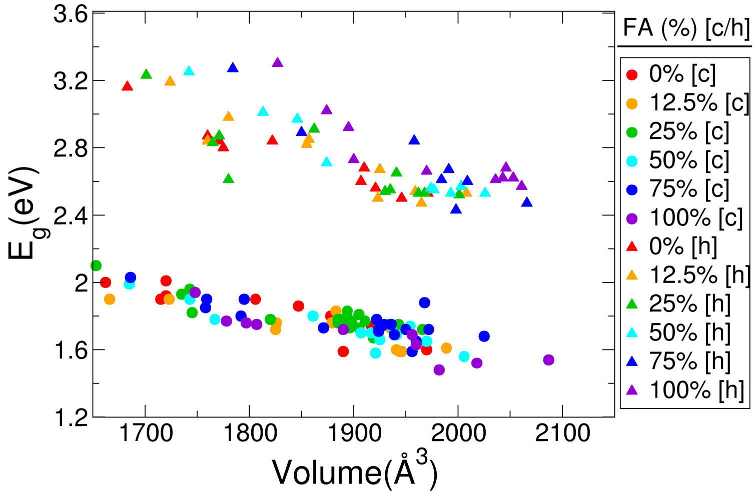

Figure S10: Correlation between volume and band gap of the hexagonal or cubic $FA_xMA_{1-x}Pb[Br_yI_{1-y}]_3$ hybrid perovskite supercell with the arrangement of (a) aligned and (b) rotated organic molecules. The colours differentiate the proportion of MA to FA.

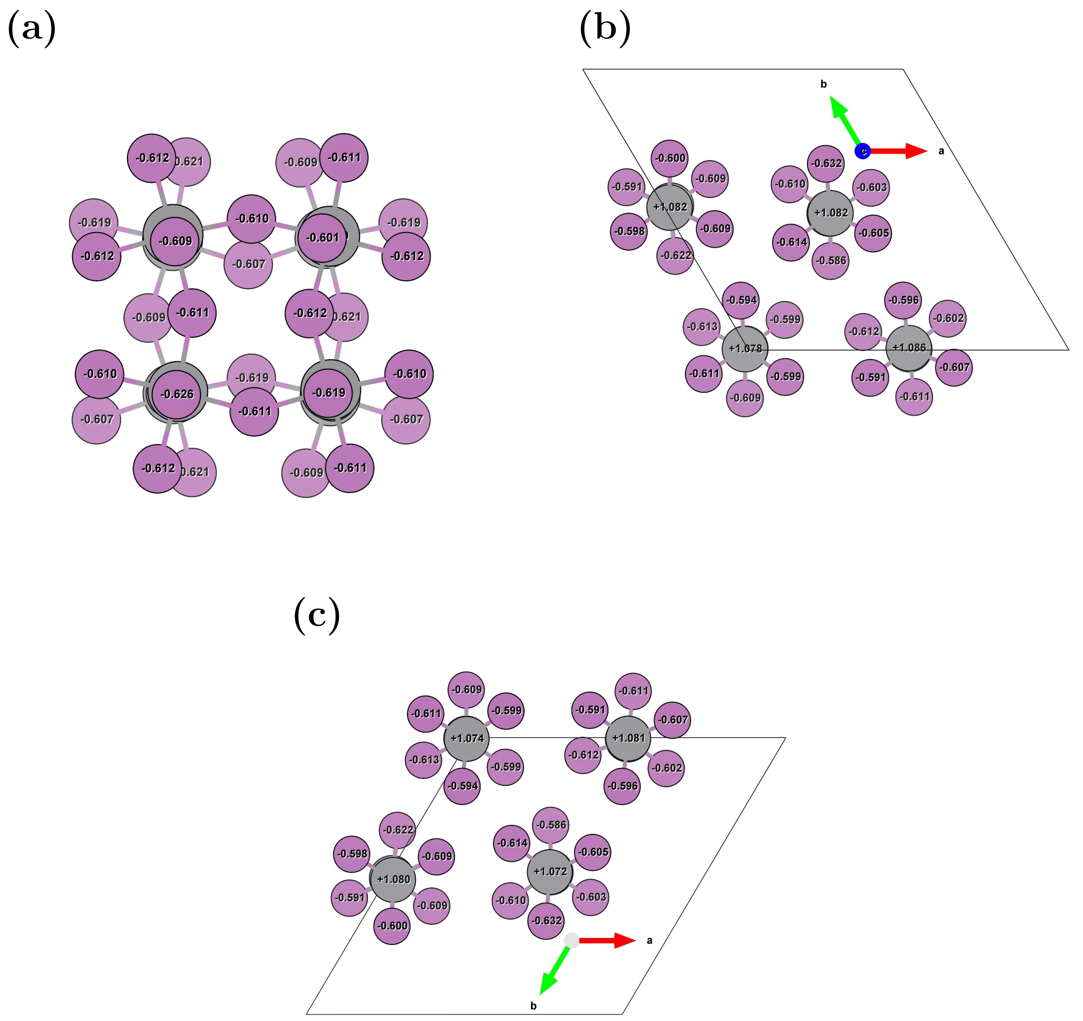

Figure S11: Bader net charges of Pb and I atoms labelled in the geometrical structures (a) cubic-like MAPI and hexagonal MAPI (b) and (c).

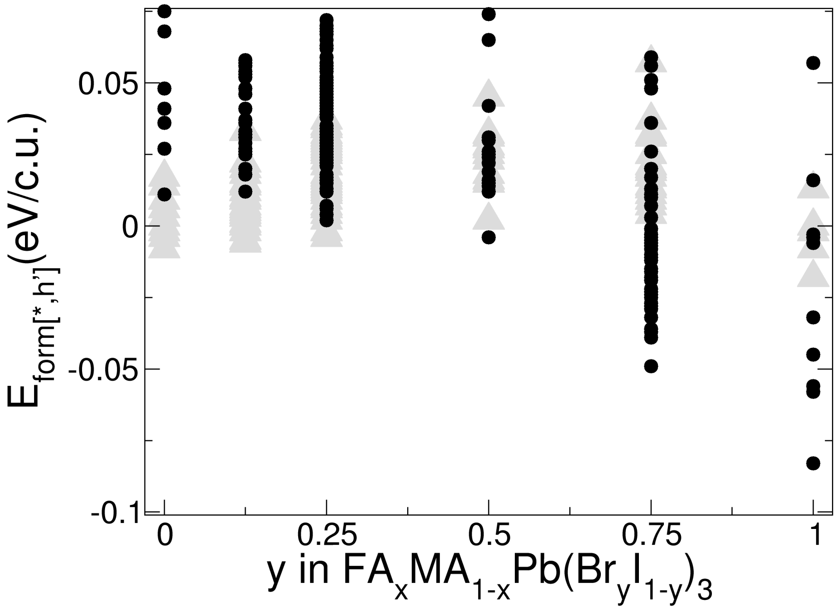

Figure S12: Formation energy distribution of perovskites in cubic-like (black) and hexagonal phases (grey).

(a)

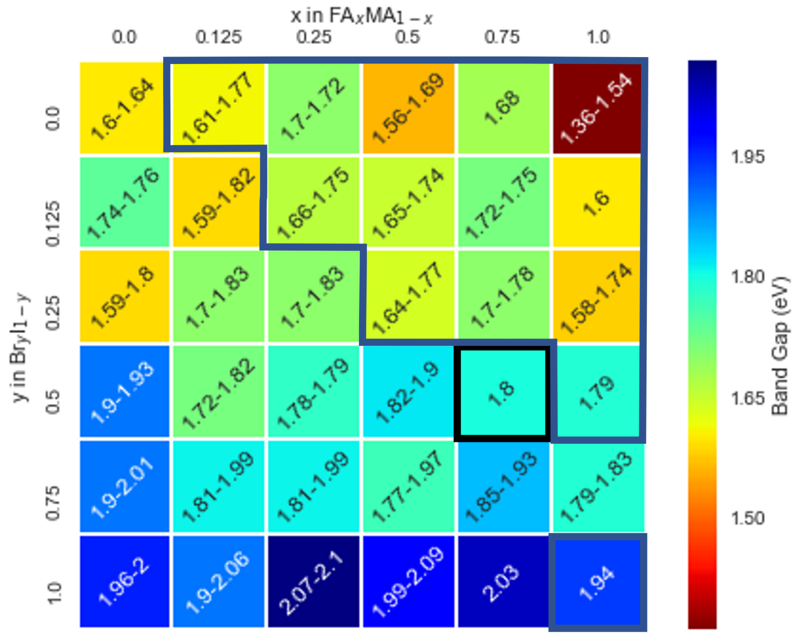

(b)

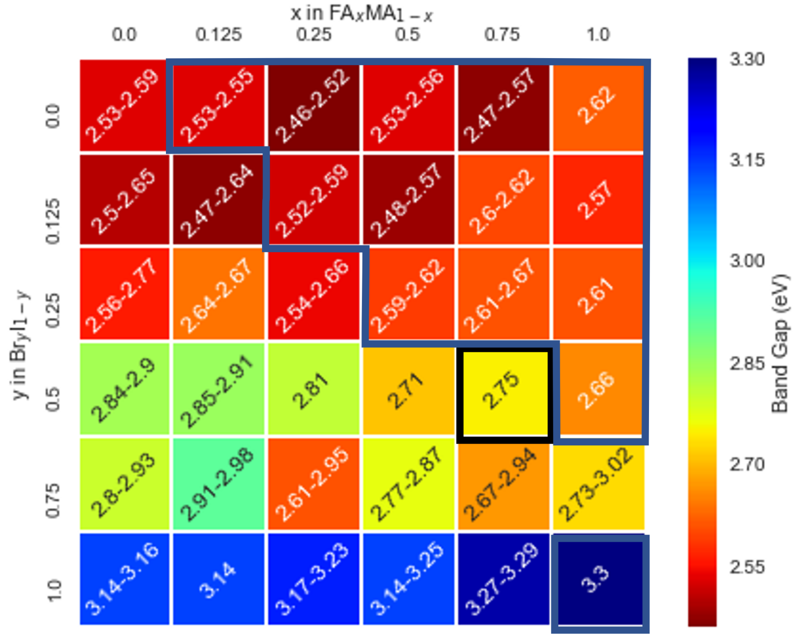

Figure S13: The variation of the band gap with different mixes of $FA_xMA_{1-x}Pb[Br_yI_{1-y}]_3$ in (a) cubic-like phase and (b) hexagonal phase.

(a)

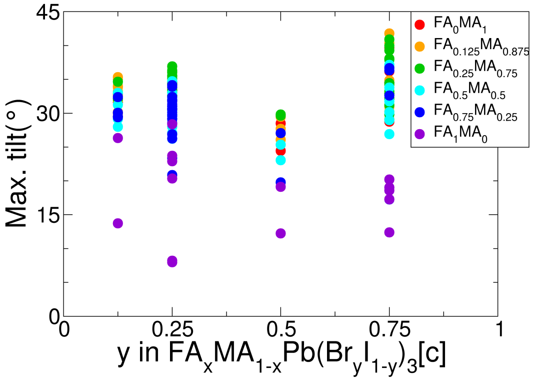

(b)

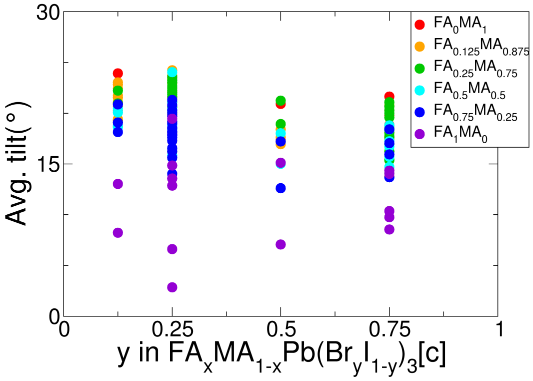

(c)

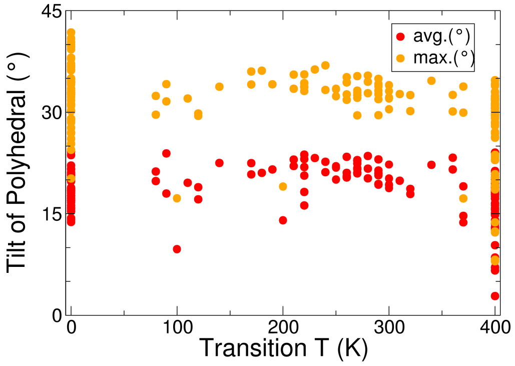

Figure S14: Distribution of tilt angle of PbX_6 polyhedral versus (a) & (b) composition and (c) transition temperature in cubic-like phase perovskite.
